# Pre- and post-copulatory traits are affected by experimental inbreeding, but they are not correlated

**DOI:** 10.1186/s12915-025-02245-4

**Published:** 2025-05-28

**Authors:** Doris Nicolakis, Maria Adelaide Marconi, Kerstin E. Auer, Dustin J. Penn, Sarah M. Zala

**Affiliations:** 1 Department of Interdisciplinary Life Sciences, Konrad Lorenz Institute of Ethology, University of Veterinary Medicine Vienna, Vienna, Austria; 2 Department of Biological Sciences and Pathobiology, Institute of in Vivo and in Vitro Models, University of Veterinary Medicine Vienna, Vienna, Austria

**Keywords:** Sexual signals, Inbreeding depression, Allocation tradeoffs, Ultrasonic vocalizations, Sperm traits, Wild house mice, *Mus musculus musculus*

## Abstract

**Background:**

It has been suggested that the expression of males’ secondary sexual traits provides reliable indicators of their sperm traits, predicting positive correlations between pre- and post-copulatory traits (Fertility Indicator Hypothesis). Yet, it has also been suggested that males face life-history tradeoffs between investing into primary versus secondary sexual traits, predicting negative correlations (Sexual Allocation Tradeoff Hypothesis). These two hypotheses are not mutually exclusive when males’ sexual traits are condition-dependent and high-quality males are better able to invest into both pre- and post-copulatory traits than low-quality males. To test these hypotheses, we manipulated the genetic quality of wild-derived male house mice by experimental inbreeding and first tested whether inbreeding affects primary or secondary sexual traits (condition-dependent expression). We then tested whether pre- and post-copulatory traits are correlated. We recorded courtship behavior and vocalizations of the males during female contact and measured males’ reproductive organs, sperm quality, and the expression of four genes associated with spermatogenesis.

**Results:**

Inbreeding did not reduce male courtship vocalizations, though it altered their vocal repertoire and reduced other courtship behaviors. Inbreeding negatively impacted relative testes mass and sperm quantity and quality, after two generations of inbreeding. We found no consistent correlations between pre-and post-copulatory traits, either positive or negative, regardless of inbreeding.

**Conclusions:**

Our results indicate that inbreeding impacted the expression of primary and secondary sexual traits in wild-derived house mice, which is the first such evidence to our knowledge, but we found no support for either the Fertility Indicator or the Sexual Allocation Tradeoff Hypotheses.

**Supplementary Information:**

The online version contains supplementary material available at 10.1186/s12915-025-02245-4.

## Background

**Fig. 1:**
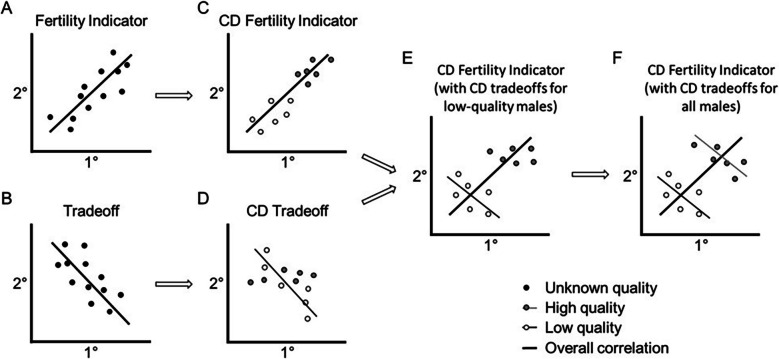
Graphical illustration of the theoretical predictions for the relationships between the expression of males’ primary (1°) post-copulatory versus secondary (2°) pre-copulatory sexual traits, and how they are expected to depend on male condition (the overall relationship is shown as a thick black line, with the high-quality males shown in grey circles versus low-quality males in white circles). The expressions of primary and secondary sexual traits have been predicted to be (**A**) positively correlated (Fertility Indicator Hypothesis) and (**B**) negatively correlated (Sexual Allocation Tradeoff Hypothesis). Positive correlations could be explained by (**C**) differences in male condition (Condition-Dependent (CD) Fertility Indicator), and (**D**) tradeoffs may be condition-dependent, with only low-quality males facing tradeoffs. If the overall relationship is positive due to differences in male condition (as with C), then it is also possible that (**E**) low-quality males or (**F**) all males face tradeoffs. Such predications have been proposed to explain why the expected life-history tradeoffs are often not found (due to variation among genotypes in acquiring resources) [4], and these insights also suggest how two seemingly mutually exclusive (or opposite) predictions can be resolved

It has been suggested that the expression of male secondary sexual traits provides honest or reliable indicators of males’ fertility, and that females use these traits to avoid mating with infertile males (fertility indicator or “phenotype-linked fertility” hypothesis, [1]). This hypothesis predicts that the expression of male secondary sexual (pre-copulatory) traits is positively correlated with primary sexual (post-copulatory) traits, such as sperm count and testes size (Fig. 1A). Yet, it has also been proposed that males face an allocation tradeoff between investing into the development of primary versus secondary sexual traits, which predicts that these traits are negatively correlated (sexual allocation tradeoff) [2] (Fig. 1B). Previous studies provide empirical evidence for both predictions, as some studies find positive correlations, whereas others find a negative or no correlation between these traits [3]. Variation in male quality or condition may mask tradeoffs and even generate positive correlations among life-history traits (“Y-model of resource allocation tradeoffs” or “big houses, big cars” hypothesis [4–6]) (Fig. 1C and D), which suggests that, unlike males in poor condition, high quality males may invest into both primary and secondary sexual traits - and without facing a tradeoff. If so, then we would expect that male secondary sexual signals provide honest condition-dependent indicators of fertility (overall positive correlation), and if there are tradeoffs, then these will be more likely to occur in low- than high-quality males (Fig. 1E versus F). Thus, we expect that both positive and negative trends can appear once we control for male condition (see Simpson’s paradox [7–9]).

We aimed to test these predictions in wild-derived male house mice (*Mus musculus musculus)* by manipulating male condition with experimental inbreeding and then analyzing the relationships between male pre- and post-copulatory traits and their interactions. House mice emit vocalizations during courtship and mating mainly in ultrasonic (> 20 kHz) frequency ranges [10–13], but also in sonic (< 20 kHz) frequencies [14–17]. During opposite-sex interactions, approximately 85% to 96% of all detected ultrasonic vocalizations (USVs) are emitted by the males [18–21], whereas sonic squeaks are suspected to be emitted mainly by females [15, 22]. Male USVs have been proposed to have several signaling functions, which include signaling sexual motivation [10, 16], facilitating courtship and mating [13], as well as mediating social recognition [23–25]. Male USV emission has long been suggested to provide an honest indicator of their sexual arousal [10], and indeed, vocalization rates, vocal repertoire and USV length are increased by sexual priming (recent exposure to a female) [16]. Furthermore, different USV parameters have been shown to correlate with copulatory success (vocal repertoire, number of short syllables, mean frequency and inter-syllable interval [26]) and reproductive success (laboratory mice: number of USVs [27, 28]; wild mice: USV length and number of simple syllable types [29]). It is not known, however, whether any of these candidate parameters of male USV emission provides an honest indicator of male sperm quantity or quality (positive correlation), or whether male mice face a tradeoff between investing into secondary versus primary sexual traits (negative correlation). It is also unclear whether male USV emission is condition-dependent, though one study found that it is altered during sickness (reduced emission of “regular syllables” (frequencies between 30 and 110 kHz) and increased emission of “high syllables” (frequencies > 110 kHz)) [30].

There is no consensus for how to measure or manipulate quality or condition [31–34], and though the expression of sexual traits is often correlated with various measures of condition (such as nutrition stage, body mass or health) (reviewed in [34]), these manipulations of environmental influences on quality usually result only in short-term effects. In contrast, manipulating male genetic quality, such as through genetic engineering [35] or experimental inbreeding, provides a method to influence long-term effects at all stages of the life cycle, including development, survival, and reproductive success [36–38]. Inbreeding has a variety of harmful effects (inbreeding depression) due to homozygosity allowing the expression of recessive deleterious alleles [36, 39–41]. Studies on many species have found that inbreeding reduces the expression of primary sexual traits, including impairing sperm quantity, quality, and sperm competitiveness [42–44] and also secondary sexual traits, such as pheromone production [45]. Furthermore, inbreeding has been shown to affect different aspects of courtship vocalizations such as song phonetics in *Drosophila montana* or song sparrows (*Melospiza melodia*) [46, 47], syllable phonetics in canaries (*Serinus canaria)* [48], and call structure in field crickets (*Teleogryllus commodus*) [49]. In house mice, inbreeding reduces disease resistance [50], longevity, and male social status and reproductive success [51]. Yet, few studies have experimentally manipulated inbreeding to test its effects on the expression of primary and secondary sexual traits, and the expected allocation tradeoffs between pre- versus postcopulatory traits.

We manipulated the genetic quality of wild-derived male house mice by breeding mice for one or two generations of close inbreeding (brother-sister matings) to generate three levels of genetic quality [34, 41]. We examined the effects of inbreeding on pre-copulatory (courtship behavior and courtship vocalizations) and post-copulatory sexual traits (reproductive organs, sperm production, and gene expression associated with spermatogenesis). We expected inbreeding to have a negative impact on pre- and post-copulatory traits (condition-dependent expression). We then tested whether pre- and post-copulatory sexual traits show a positive (Fertility Indicator Hypothesis) or negative correlation (Allocation Tradeoff Hypothesis), and whether any such correlations depended upon the level of inbreeding (Fig. 1).

## Results

### Pre-copulatory traits

Our first aim was to test whether inbreeding alters courtship behavior and vocalizations emitted by males.

#### Ultrasonic vocalizations and other courtship behaviors

**Fig. 2:**
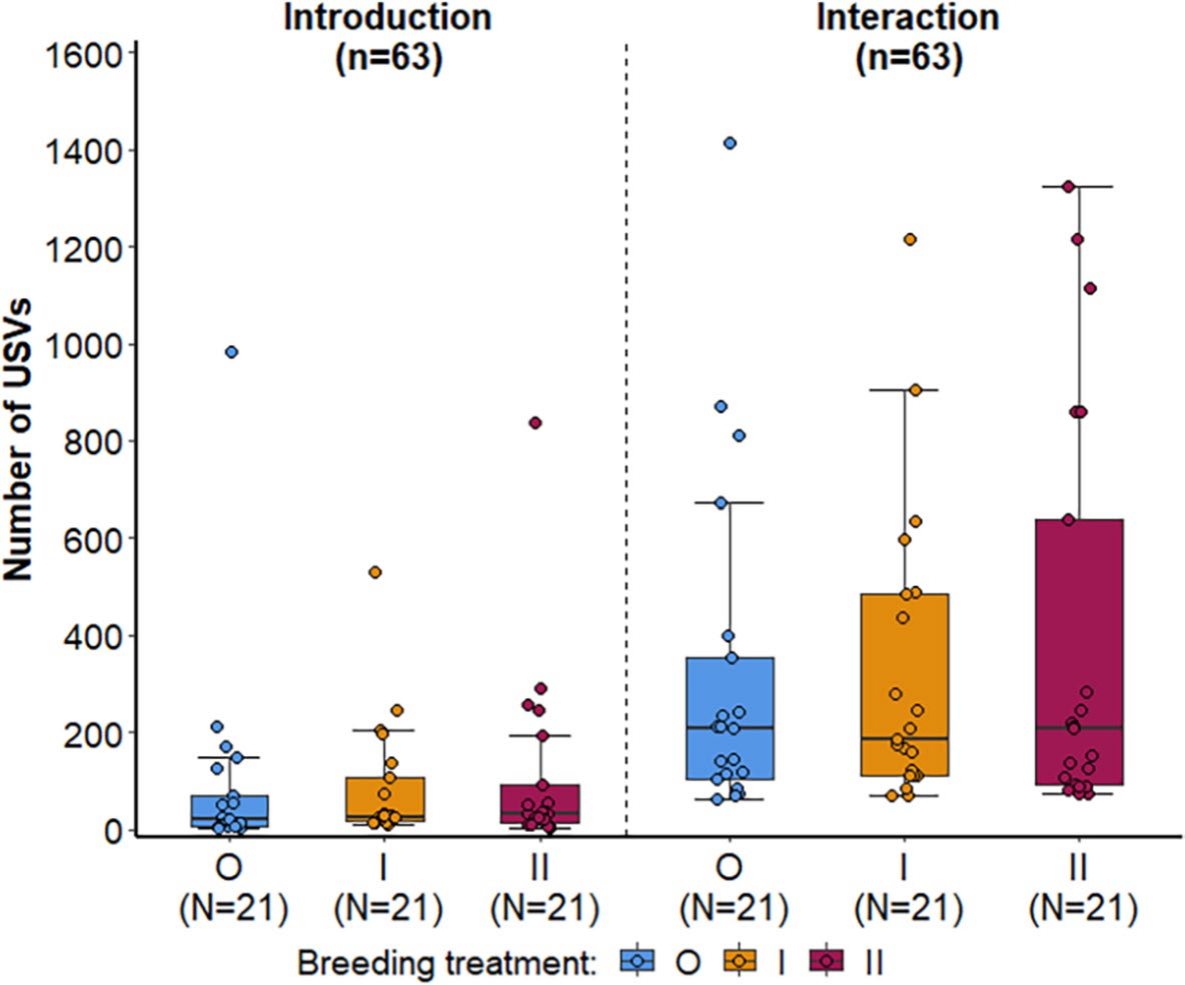
USV emission of mice during the introduction and interaction phase comparing the three breeding treatments. Points represent raw data, boxplots show median (center line), interquartile range (IQR, box), and 1.5x IQR (whiskers) of the USV count emitted by outbred (O, blue), 1^st^ generation inbred (I, orange) and 2^nd^ generation inbred (II, red) mice (shown from left to right, respectively)

As expected, the mice emitted significantly more USVs and a larger vocal repertoire during direct interactions than during the introduction phase (GZLMM: USV count, F = 49.895, *p* < 0.001, vocal repertoire, F = 23.114, *p* < 0.001) (Fig. 2); however, the three treatment groups did not differ in the USV count, the vocal repertoire (GZLMM: USV count, F = 0.427, *p* = 0.654, vocal repertoire, F = 1.373, *p* = 0.257, Fig. 2, Additional file 1: Table S4, Fig. S3), and the number of short, simple or complex USVs in either phase (Additional file 1: Table S5).

During the introduction phase, the number of USVs that males emitted positively correlated with the time that they spent at the divider (r_s_ = 0.430, *p* < 0.001, Additional file 1: Fig. S4A) and with the time spent investigating females through the divider (r_s_ = 0.350, *p* = 0.005, Additional file 1: Fig. S4B), though, when analyzing the treatment groups separately, surprisingly, these effects were only significant for O and II mice, respectively. During the interaction phase, the number of USVs correlated with the number of female defensive behaviors when pooling all males (r_s_ = 0.262, *p* = 0.038, Additional file 1: Fig. S4C). Additionally, the number of USVs was correlated with the number of female-directed investigatory behaviors, though only in outbred males; inbred males showed no such relationship (O-males, r_s_ = 0.588, *p* = 0.005, Additional file 1: Fig. S4D). Thus, the slopes of the correlations between number of USVs and female-directed investigatory behavior differed significantly between I and O mice (GLM: interaction O vs I, T = −2.693, *p* = 0.009).

**Fig. 3:**
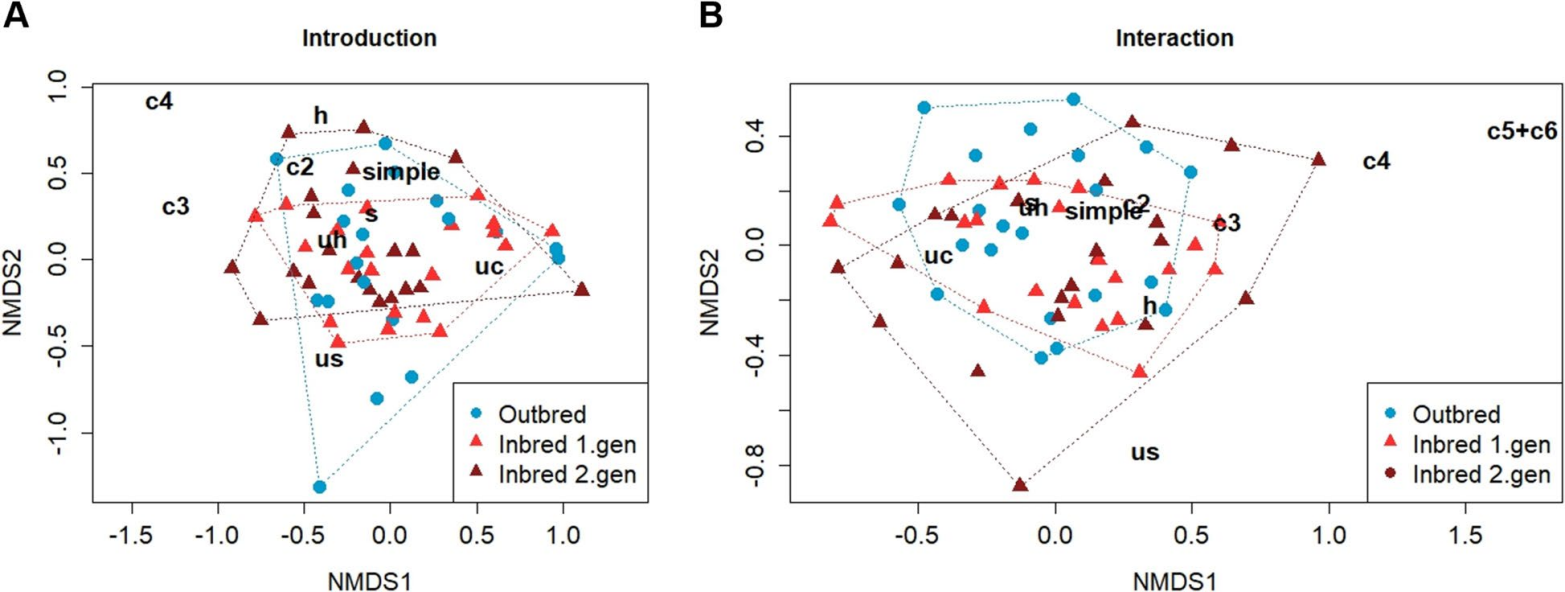
Variation in USV repertoire composition shown with non-metric multidimensional scaling (nMDS) plots during (**A**) introduction phase (using 10 syllable types) and (**B**) interaction phase (using 10 syllable types) comparing outbred (blue dots), 1^st^ generation inbred (red triangles) and 2^nd^ generation inbred (dark red triangles) mice. Letters in black indicate the syllable types and each symbol represent one recorded pair (i.e., a male from one of the 3 genetic backgrounds and an outbred female). Distances between the symbols represent similarities of pairs in the syllable type usage. Short distances of symbols to letters indicate syllable types that were most representative for each pair. Note syllable types “c5 + c6” were not emitted in the introduction phase, and thus are not presented in **A**

The USV repertoire composition did not differ among the three breeding treatments in either phase (ANOSIM: Introduction, 10 syllable types, R = 0.003, *p* = 0.409; Interaction, 10 syllable types, R = −0.025, *p* = 0.576, Fig. 3). Yet, when pooling the two inbred treatments, the repertoire composition differed significantly from the outbred males during the introduction, though not the interaction phase (ANOSIM: introduction, 10 syllable types, R = 0.086, *p* = 0.047; interaction, 10 syllable types, R = −0.017, *p* = 0.608, Additional file 1: Fig. S5A and B). These results did not differ when using all 15 syllable types for the introduction phase (see Additional file 1: Fig. S5C and D).

**Fig. 4:**
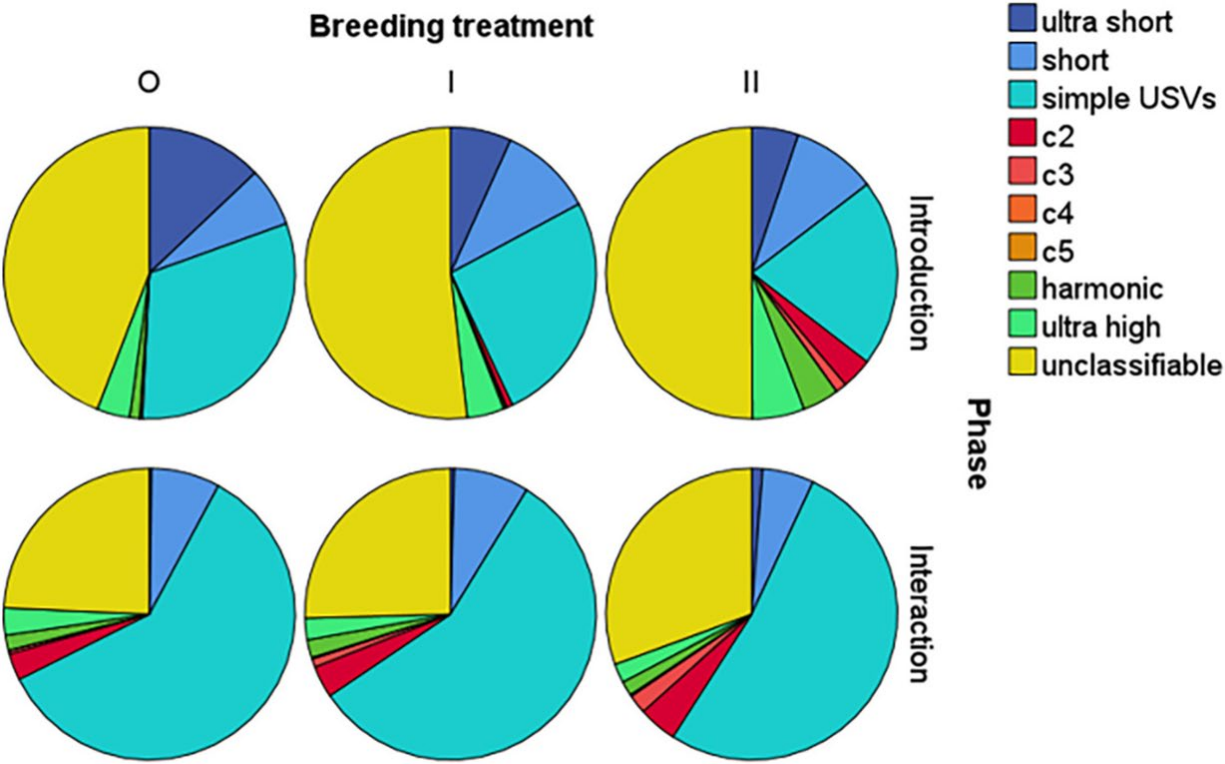
Pie charts representing proportions of each of 10 syllable types used by outbred (O), 1^st^ generation inbred (I) and 2^nd^ generation inbred (II) mice during the introduction and interaction phase. Syllable types in the legend (from top to down) refer to single sections in the pie chart clockwise starting at the top

Visual inspection of pie charts (Fig. 4) revealed that all groups emitted ca. 50% unclassifiable (“uc”) USVs during the introduction phase, whereas they emitted mainly simple USVs during direct interactions (Fig. 4). The mice emitted a higher number of short (“s”) and ultrashort (“us”) USVs during introduction compared to the interaction phase, whereas the number of complex (“c2, “c3” “c4,” and “c5”), harmonic (“h”) and ultrahigh (“uh”) USVs was generally low in both phases. During introduction, outbred males emitted more ultra short (< 5 ms) and simple syllable types (consisting of one element) compared to both inbred groups. Additionally, 2^nd^ generation inbred (II) males emitted a larger proportion of complex (consisting of 2 or more elements) and harmonic syllable types than other males (Fig. 4).

Next, we investigated the effects of inbreeding on USV spectro-temporal features. First, we used average values calculated for all USVs recorded during each phase (*n* = 63 males), which showed that mice emitted longer USVs during direct interactions compared to the introduction phase (GZLMM: F = 55.423, *p* < 0.001); but breeding treatment had no effect on mean USV length (GZLMM: F = 0.885, *p* = 0.415, Additional file 1: Table S4, Fig. S6A). There was no effect of phase or breeding treatment on the inter-syllable interval (ISI) (GLZMM, phase, F = 0.853, p = 0.358, treatment, F = 0.265, *p* = 0.768; Additional file 1: Table S4, Fig. S6C). The mice emitted USVs with a shorter inter-bout interval (IBI) during direct interactions compared to the introduction phase (GLZMM: F = 56.489, *p* < 0.001, Additional file 1: Table S4,), but the breeding treatment had no effect on the inter-bout interval (GLZMM, F = 0.927, *p* = 0.399, Additional file 1: Table S4, Fig. S6D). Furthermore, the grand mean frequency did not differ significantly between the 2 phases (GZLMM: F = 1.399, *p* = 0.239) or between treatments in either phase (GLZMM: F = 0.190, *p* = 0.827, Additional file 1: Table S4, Fig. S6B).

**Fig. 5:**
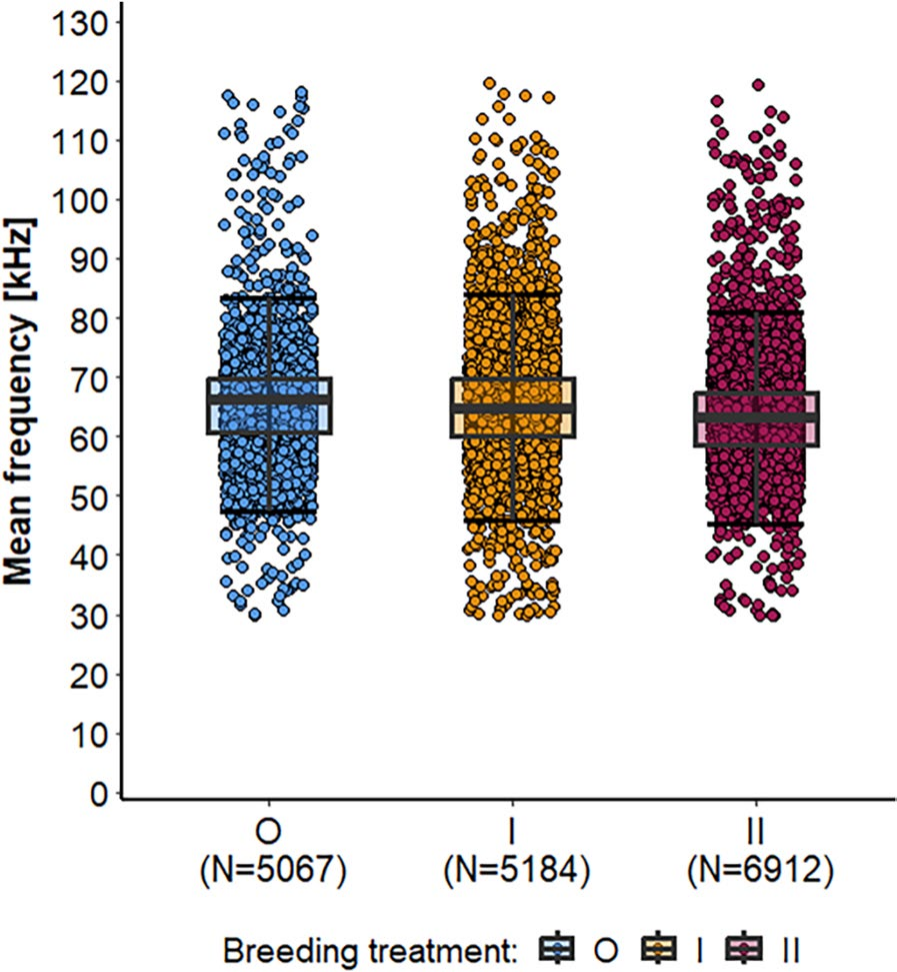
Mean frequency of each USV emitted during the interaction phase comparing 3 breeding treatments. Points represent raw data, boxplots show median (center line), interquartile range (IQR, box), and 1.5x IQR (whiskers) of USVs emitted by outbred (O, blue), 1^st^ generation inbred (I, orange) and 2^nd^ generation inbred (II, red) mice (shown from left to right, respectively)

Second, we analyzed spectro-temporal parameters using single datapoints extracted for each USV emitted during the interaction phase (*n* = 17 636 USVs). When analyzing the mean frequency, we found that the breeding treatment model was better than the null model and showed a significant treatment effect on the mean frequency (Fig. 5, Additional file 1: Table S6). Post-hoc comparisons with adjusted p-values showed that inbred (I) mice tended to emit USVs at a lower mean frequency than outbred males (mean ± sd, median: O, 65.4 ± 8.2 kHz, med = 66.2 kHz; I, 65.4 ± 9.7 kHz, med = 64.6 kHz; z = 2.053, *p* = 0.099), but at a higher mean frequency than II males (mean ± sd, median: II, 63.5 ± 8.4 kHz, med = 63.2 kHz; z = -2.279, *p* = 0.058).

Similarly, when analyzing the inter-syllable interval (ISI), the breeding treatment model was better than the null model showing a significant treatment effect on the inter-syllable interval (ISI) when using single datapoints (Additional file 1: Table S6). Post-hoc comparisons with adjusted *p*-values showed that I males emitted USVs with the longest ISI compared to both O and II males (mean ± sd, median: O: 111.74 ± 68.43 ms, med = 79.6 ms; I, 117.7 ± 66.48 ms, med = 87.0 ms; II: 113.1 ± 68.84 ms, med = 80.3 ms; I vs. O: z = −3.478, *p* = 0.002, I vs. II: z = 2.928, *p* = 0.01). The results of USV length and IBI did not change when using single datapoints for analyses, where the treatment model was not better than the null model, showing that breeding treatment had no significant effect (Additional file 1: Table S6).

#### Sonic vocalizations and other courtship behaviors

**Fig. 6:**
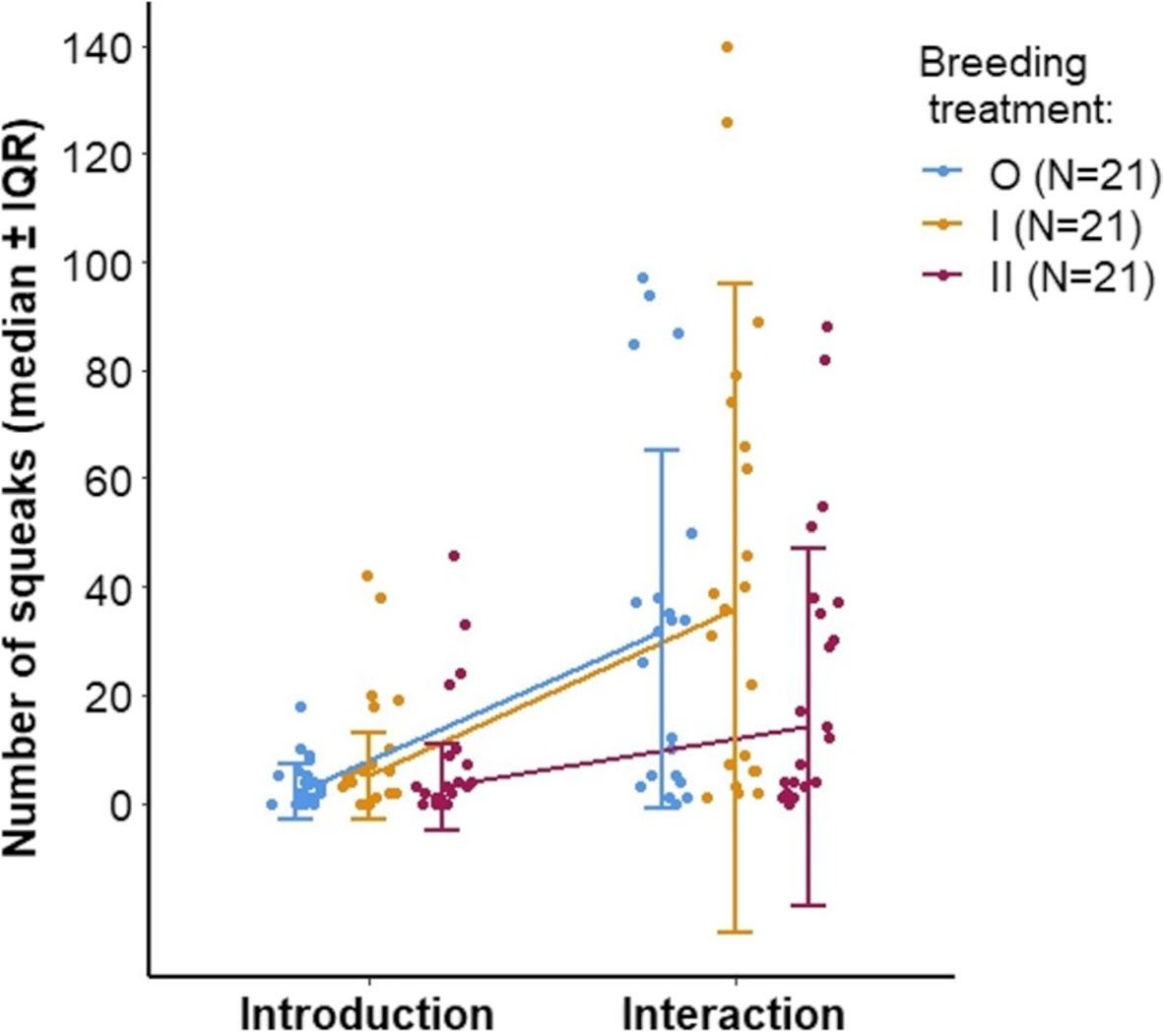
Numbers of squeaks during the introduction versus interaction phase, comparing 3 breeding treatments (outbred mice (O): blue, left; 1^st^ generation inbred (I): orange, middle; and 2^nd^ generation inbred mice (II): red, right). Points represent raw data, line graphs presenting medians ± interquartile range (IQR), showing the interaction between phase and treatment group

The mice emitted more mid-frequency vocalizations (MFVs < 20 kHz) during the introduction phase, whereas they emitted more “squeaks” (sonic vocalizations with multiple harmonic elements, F_0_ < 20 kHz) during the interaction phase (Additional file 1: Table S4, Fig. S7). The number of MFVs and squeaks did not differ between the three breeding treatments regardless of the recording phase (GZLMM: MFV, F = 0.351, *p* = 0.705, squeaks, F = 2.038, *p* = 0.135, Additional file 1: Table S4, Fig. S7). However, the number of squeaks tended to show an interaction between phase and treatment. Whereas all 3 treatment groups emitted a comparable number of squeaks during the introduction phase, II mice emitted fewer squeaks compared to the other 2 groups during the interaction phase (Fig. 6, phase * breeding treatment interaction: GZLMM: F = 2.368, *p* = 0.098, Additional file 1: Table S4).

**Fig. 7:**
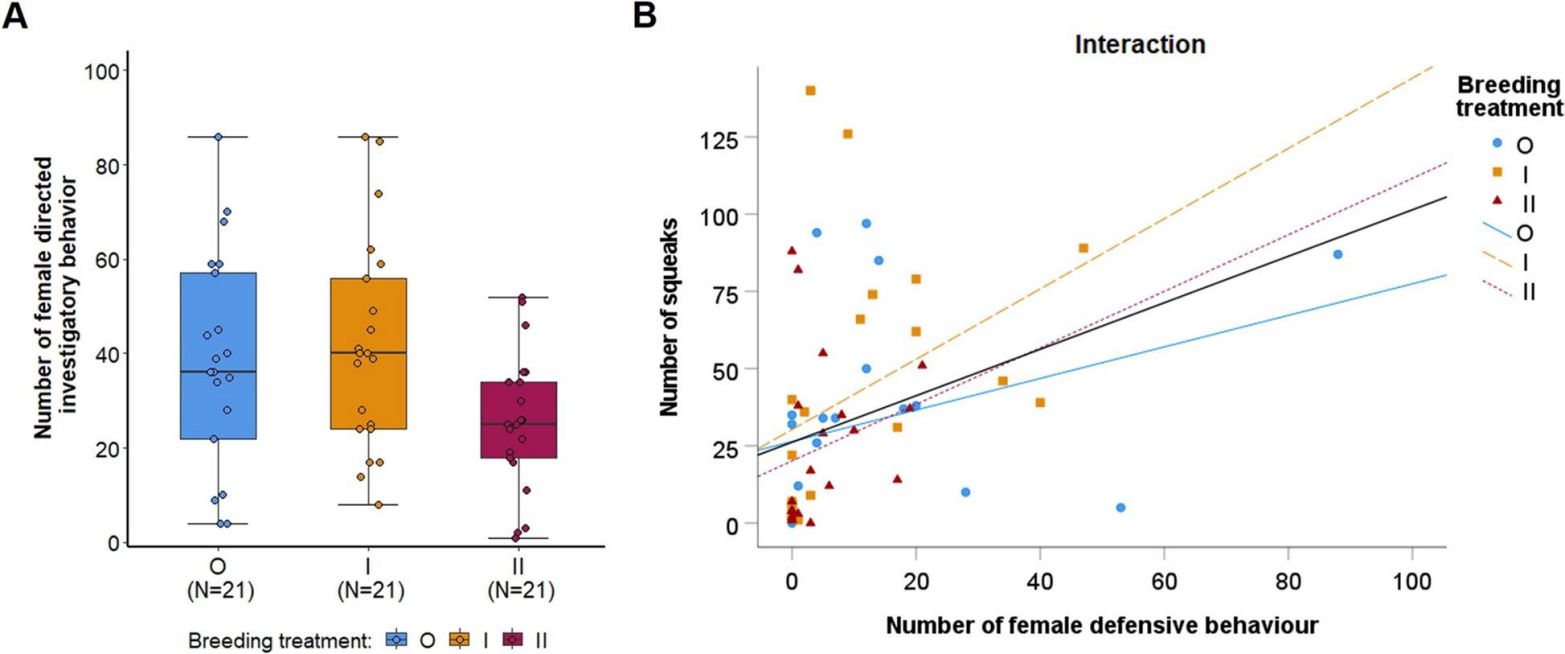
**A** Number of investigatory behaviors of males towards females comparing the three breeding treatments. Points represent raw data, boxplots show median (center line), interquartile range (IQR, box) and 1.5x IQR (whiskers). **B** Correlation between number of female defensive behaviors and number of squeaks. Symbols represent outbred (O, blue circles, solid line), 1^st^ generation inbred (I, orange squares, dashed line), and 2^nd^ generation inbred (II, red triangles, dotted line) males. The black line represents the overall correlation

Similarly, highly inbred (II) males showed less investigatory behavior towards females during direct interactions compared to outbred (O) and 1^st^ generation inbred (I) males (Kruskal Wallis test: *n* = 63, H = 6.354, *p* = 0.0042) (Fig. 7A). Overall, the number of squeaks emitted during direct interactions was positively correlated with the total duration of males’ female-directed investigatory behavior (r_s_ = 0.270, *p* = 0.032) and with the number of female defensive behaviors (r_s_ = 0.611, *p* < 0.001, Fig. 7B). Additionally, the number of female defensive behaviors correlated with the number of female-directed investigatory behaviors (r_s_ = 0.404, *p* = 0.001).

### Post-copulatory traits: sperm traits, gene expression and reproductive organs

**Fig. 8:**
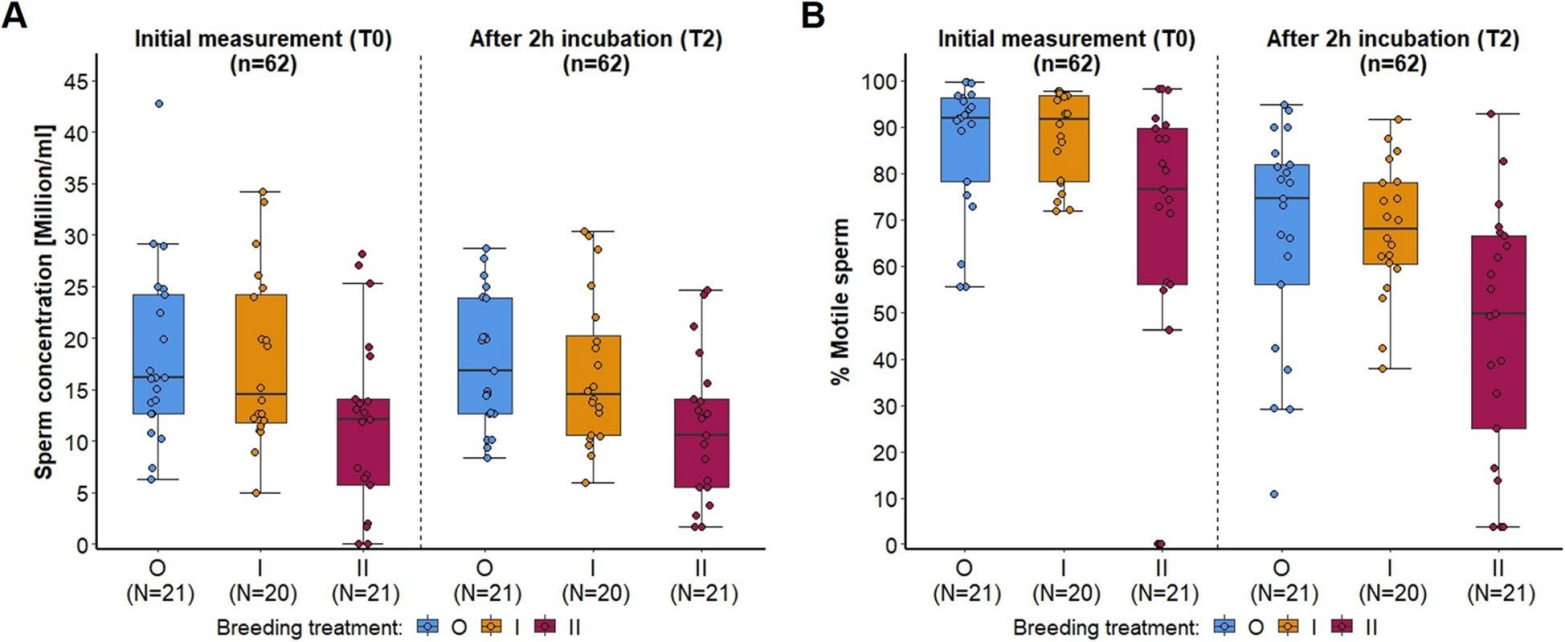
Sperm quantity and quality measured during two timepoints (initial measurement immediately after sperm collection (T0), and after a 2 h incubation period (T2)) for the three breeding treatments. Points represent raw data, boxplots show median (center line), interquartile range (IQR, box), and 1.5x IQR (whiskers) of (**A**) sperm concentration (million/ml) and (**B**) percentage of motile sperm of outbred (O, blue), 1^st^ generation inbred (I, orange) and 2^nd^ generation inbred (II, red) males (shown from left to right, respectively)

Our second aim was to investigate whether inbreeding influences sperm traits, gene expression related to spermatogenesis, or reproductive organs. We found that inbreeding significantly reduced sperm concentration (GZLMM: F = 5.291, *p* = 0.006, Additional file 1: Table S7; Fig. 8A) and the percentage of motile sperm (GZLMM: F = 7.431, *p* = 0.001, Additional file 1: Table S7; Fig. 8B). Pairwise comparisons showed that II mice produced significantly less sperm and with a lower percentage of motile sperm compared to both other groups (Fig. 8). The percentage of motile sperm was significantly reduced after 2-h incubation (T2) in all groups, and the model showed no interaction between the treatment and the time point of sperm measurement (Additional file 1: Table S7).

**Fig. 9:**
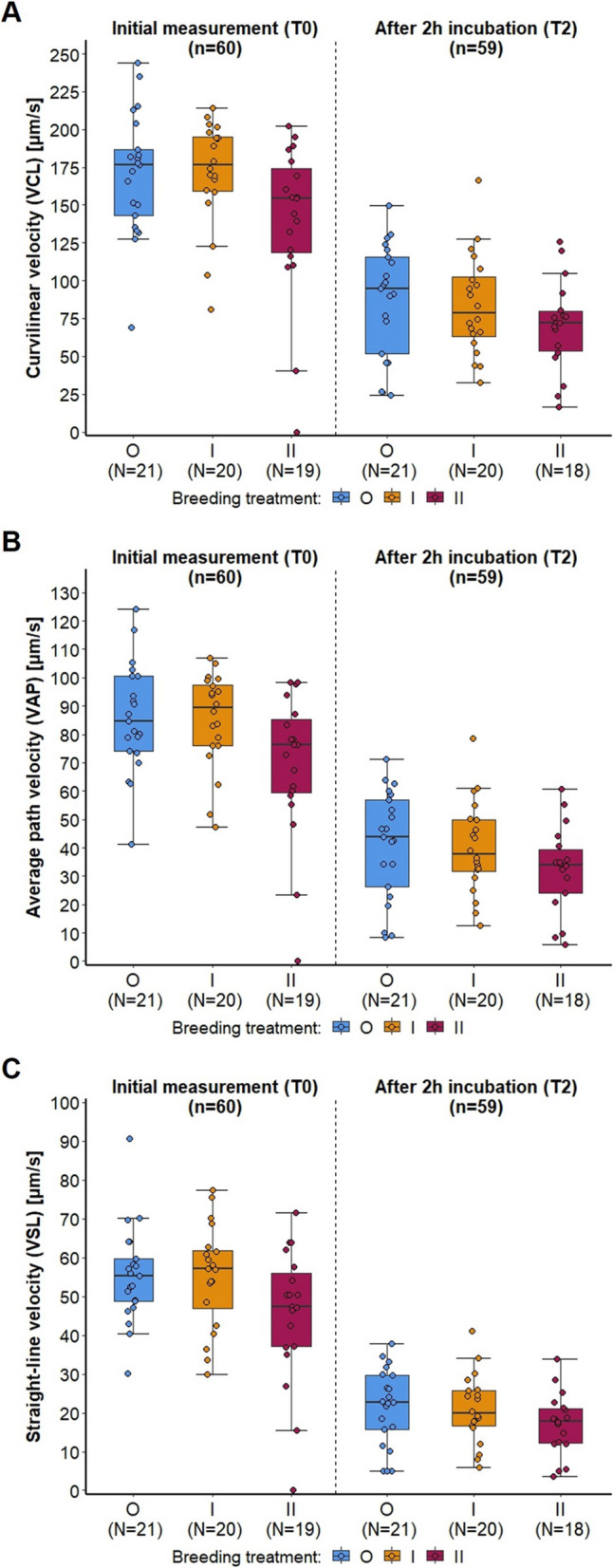
Sperm quality measured during two timepoints (initial measurement immediately after sperm collection (T0), and after a 2-h incubation period (T2)) for the three breeding treatments. Points represent raw data, boxplots show median (center line), interquartile range (IQR, box), and 1.5x IQR (whiskers) of (**A**) curve speed (curvilinear velocity, VCL, µm/s), (**B**) average speed (average path velocity, VAP, µm/s), and (**C**) linear speed (straight-line velocity, VSL, µm/s) of sperm from outbred (O, blue), 1^st^ generation inbred (I, orange) and 2^nd^ generation inbred (II, red) males (shown from left to right, respectively)

Inbreeding also had a significant effect on VCL (curvilinear velocity), VSL (straight linear velocity) and VAP (average path velocity), and these parameters significantly declined over time (in 2 h from T0 to T2) (GZLMM: all *p* < 0.001, Additional file 1: Table S7; Fig. 9). During T0 but not T2 measurement, the sperm of II mice had lower VAP and VSL and tended to have lower VCL, compared to both other groups (Fig. 9; Additional file 1: Table S7). There were no interactions between the time points and the breeding treatment.

Even though inbreeding affected sperm traits, we found no significant effect of inbreeding on the expression of four target genes (Protamine-2, GAPDS, Park-2 and STK22B) which are associated with spermatogenesis and male fertility (Additional file 1: Table S8, Fig. S8). Furthermore, there was no significant correlation between gene expression and sperm concentration, motility or sperm velocity parameters at T0, respectively, over all treatment groups, and results of correlations for each group separately were inconclusive (Additional file 1: Table S9, Fig. S9).

**Fig. 10:**
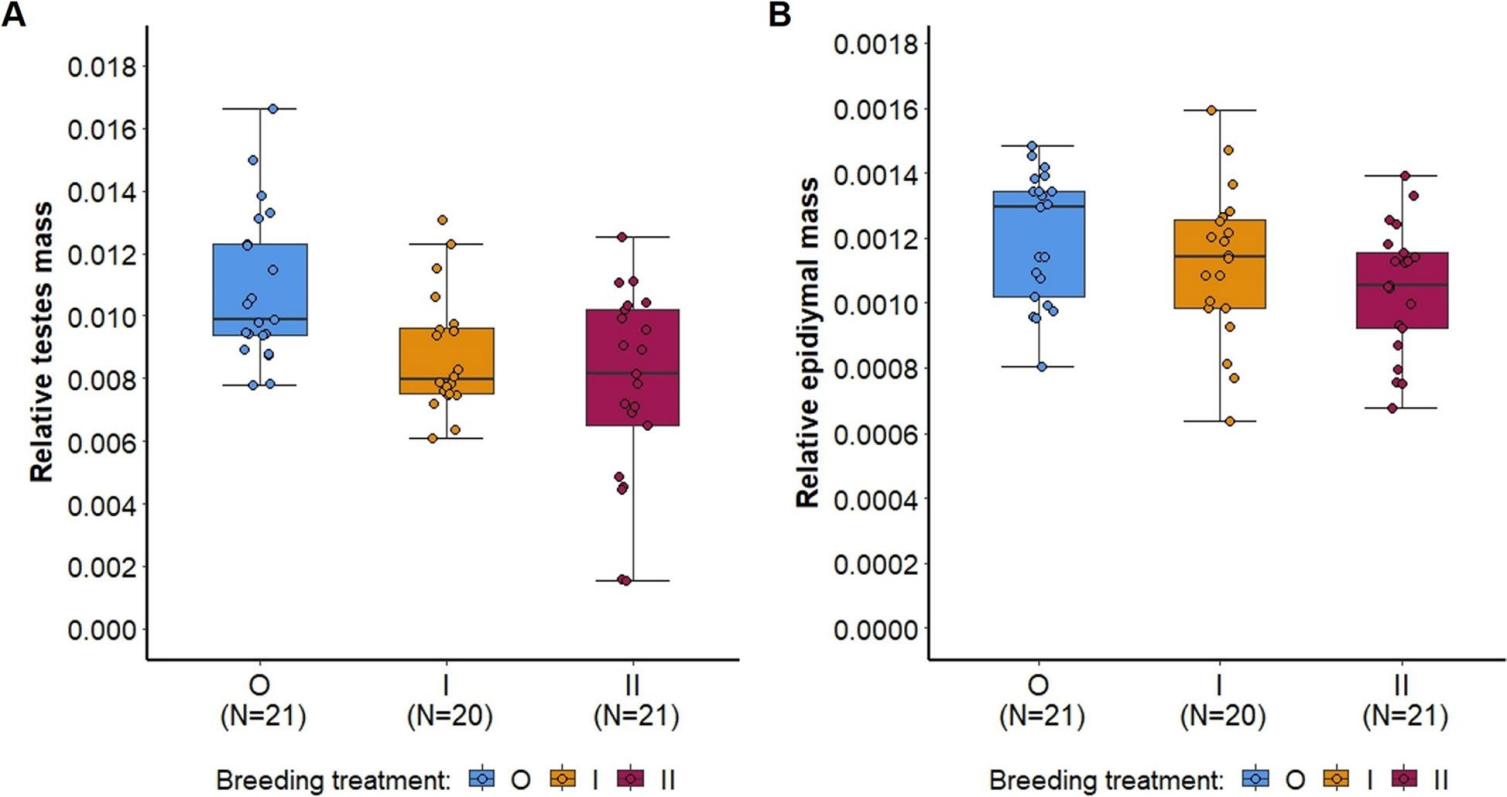
Reproductive organs corrected for body mass shown for the three breeding treatments. Points represent raw data, boxplots show median (center line), interquartile range (IQR, box), and 1.5x IQR (whiskers) of (**A**) relative testes mass, and (**B**) relative epididymal mass of outbred (O, blue), 1^st^ generation inbred (I, orange) and 2^nd^ generation inbred (II, red) males (shown from left to right, respectively)

Inbreeding had a significant effect on relative testes mass (corrected for body mass) (ANOVA: F = 8.225, *p* = 0.001, Additional file 1: Table S8; Fig. 10A) with both inbred groups showing a significantly lower relative testes mass than outbred mice (O vs I, *p* = 0.027, O vs II, *p* = 0.001, I vs II, *p* = 0.539, Sidak correction). Additionally, the total epididymal mass differed between the groups (ANOVA: F = 3.759, *p* = 0.029, Additional file 1: Table S8), though not significantly after correcting for body mass (relative epididymal mass: ANOVA, F = 2.947, *p* = 0.06, Additional file 1: Table S8; Fig. 10B). 2^nd^ generation inbred (II) mice had a lower total epididymal mass than outbred mice (O vs II: total epididymal mass *p* = 0.024; relative epididymal mass: *p* = 0.054, Sidak correction). Inbreeding had no significant effect on total or relative seminal vesicle mass (Additional file 1: Table S8). Furthermore, we found that sperm concentration was correlated with relative testes, with epididymal mass, and with relative anogenital distance (Additional file 1: Table S9, Fig. S10).

### Correlations between pre- versus post-copulatory traits

Our third aim was to test whether USV emission was correlated with sperm production. When testing for a relationship between USV versus sperm quantity, we found no correlation between the number of emitted USVs of either phase and the sperm concentration measured at T0. Further exploratory analyses including USV and sperm quality showed that the grand mean frequency of USVs emitted during the introduction phase was negatively correlated with the sperm concentration in I mice (I, r_s_ = −0.525, *p* = 0.018, Additional file 1: Fig. S11A and B, Table S10), however, this relationship was not significant after FDR correction (*p* = 0.114). For the same group (i.e., I males), the total number of USVs emitted during direct interactions tended to be negatively correlated with the average sperm curve speed (I, r_s_ = −0.424, *p* = 0.063, FDR-corrected: *p* = 0.504, Additional file 1: Fig. S11C and D, Table S10). Since scatterplots for these variables indicated a non-linear relationship (Additional file 1: Fig. S11C and D), we conducted additional logarithmic regressions, which did not change the results. We also examined the relationship between USV emission and reproductive organs. Overall, the number of USVs, the grand mean frequency and the mean length of USVs emitted during direct interactions were negatively correlated with the relative epididymal mass (USV count, r_s_ = −0.430, *p* < 0.001, FDR-corrected: *p* = 0.007, grand mean frequency, r_s_ = −0.354, *p* = 0.005, FDR-corrected: *p* = 0.04; USV length, r_s_ = −0.370, *p* = 0.003, FDR-corrected: *p* = 0.024, Additional file 1: Table S10) and the number of USVs during direct interactions was also negatively correlated with the relative testes mass (r_*s*_ = −0.303, *p* = 0.017), though not significantly after FDR correction (*p* = 0.068, Additional file 1: Table S10).

## Discussion

This is the first study on house mice to our knowledge to provide evidence that inbreeding influences the expression of both male courtship behavior and courtship vocalizations (altering USV syllable complexity and their temporal structure and reducing mean frequency (kHz)). It is also the first study in house mice to show that inbreeding has negative impacts on primary sexual traits (reducing relative testes mass and both sperm quantity and quality). Thus, we provide the first evidence in house mice that both pre- and post-copulatory traits are condition-dependent. However, we found no consistent evidence for correlations (neither positive or negative) between pre- and post-copulatory traits overall, or within inbred or outbred males, and thus, we found no support for either the Fertility Indicator or Sexual Allocation Tradeoff Hypotheses. Below we address our main findings in more detail and we propose how future studies are needed to test the Fertility Indicator and Sexual Allocation Tradeoff Hypotheses.

### Pre-copulatory traits

We found that inbreeding significantly reduced males’ female-directed investigatory behavior, though only among the 2^nd^ generation inbred males (Fig. 7A), and that inbreeding also altered the quality, though not the quantity, of males’ courtship vocalizations. Once males began to directly interact with a female, they showed a significant increase in number and length (duration) of USVs, as expected [29]; however, inbreeding did not affect either of these vocal parameters. Yet, inbreeding altered males' vocal repertoire composition during the introduction phase (Additional file 1: Fig. S5A). Visual inspection showed that outbred mice emitted a larger proportion of ultra short and simple USVs during the introduction phase, whereas 2^nd^ generation inbred males emitted more complex types with frequency-jumps and harmonic elements (Fig. 4). Male mice mostly emit simple USVs (mainly syllable type “up”) at the beginning of courtship and then shift to producing more complex and longer USVs (long USVs with multiple jumps and harmonic elements) later, just before and during mounting attempts [12, 13, 26, 52]. If emission of USVs in this order, from mainly simple calls to mostly long complex USVs, is functional, then outbred males should have an advantage. Female mice can discriminate playbacks of simple versus complex male USVs [53, 54] and females appear to be more attracted to complex over simple USVs [55]. The effects of these types of USVs on mating success are not known, though a recent study found that the emission of short syllables and USVs at higher frequencies (kHz) during early courtship correlated with copulatory success [26]. There is mixed evidence on the effects of inbreeding on repertoire size in birds: inbreeding reduced repertoire size in song sparrows (*Melospiza melodia*) [47]; however, inbreeding had no effect on repertoire size in canaries (*Serinus canaria*) [48].

We examined females' responses to male courtship during the interaction phase, and we found that female defensive behaviors are correlated with number of squeaks, which is consistent with evidence that squeaks are emitted by females when repelling male mating attempts [15, 56, 57], though we cannot rule out the possibility that males emitted squeaks as well. Additionally, the number of squeaks is associated with males' female-directed investigatory behaviors. Since inbreeding reduced males' female-directed investigation, then this could also explain why we detected fewer squeaks (which might have been emitted by either sex) when females interacted with 2^nd^ generation inbred males. In contrast, de Boer et al. [38] found that inbred canaries (*Serinus canaria*) are more motivated to investigate mating opportunities than outbred males, potentially to counteract lower reproductive success caused by inbreeding depression. Nevertheless, direct interactions between males and females are likely to influence the partner’s behavior [15, 17, 58], and thus female-directed investigatory behaviors, female defensive behaviors and squeaks might not be independent from each other.

Our analyses of spectro-temporal vocal features indicate that the mean frequency of USVs was slightly reduced in 1^st^ and 2^nd^ generation inbred mice compared to outbred males. This result is consistent with results from canaries, where inbred individuals emitted calls at lower frequencies, especially short duration calls [48]. In contrast, in the subdesert mesite (*Monias benschi)*, longer and lower-pitched trills are emitted in the songs of more heterozygous males [59].

During direct interactions, the mice emitted longer syllables with shorter inter-bout intervals (IBI) compared to the introduction phase. Furthermore, during direct interaction 1^st^ generation inbred mice emitted USVs with longer inter-syllable interval (ISI) compared to outbred mice, though 2^nd^ generation inbred mice did not differ from outbred controls. Similarly, a study on field crickets (*Teleogryllus commodus*) showed that one generation of inbreeding significantly altered the interval between pulses within a call and the interval between calls, but not the dominant frequency of a call or calling rate [49].

Thus, our results indicate that inbreeding altered male courtship behaviors, and though it had no effect on USV numbers or length, two parameters that correlate with male reproductive success [29], it altered other USV parameters (reducing the percentage of simple versus complex types and mean frequency and increasing inter-syllable intervals), which have been found to affect male mating success [26]. Future studies aiming to evaluate the effects of male USV emission on mating success need to control for differences in female-directed investigation and other sexual behaviors among males, and also determine which sex is emitting the vocalizations (squeaks as well as USVs). Courtship is a complex and interactive process between the sexes, and recent evidence indicates that female squeaks can influence and suppress male USV emission [17, 22]. Males presented with a combination of female odor and female USVs showed a significant higher increase in their USV response compared to a presentation of female odor and squeaks [22], and males reduced their USV emission during the presentation of playbacks of female squeaks [17], suggesting that female squeaks can reduce male courtship behavior. Thus, when analyzing courtship behavior, future studies need to consider the dynamic interactions of males and females and the effects of male vocalizations on their mating success.

### Post-copulatory traits

Our results show that inbreeding negatively impacted male reproductive organs and sperm quantity and quality of male house mice, which are novel to our knowledge, and consistent with studies on other vertebrates, such as oldfield mice (*Peromyscus polionotus*) [42] and zebra finches (*Taeniopygia guttata*) [60]. Inbred males had lower relative testes mass and epididymal mass, sperm concentration was 40% lower and sperm motility was 20% lower in 2^nd^ generation inbred mice compared to outbred males. Thus, inbreeding reduced sperm number, motility and swimming performance. Relative testes and epididymal mass were positively correlated with sperm concentration, suggesting that increased reproductive organ mass explains sperm quantity. It is unclear, however, why one generation of inbreeding was sufficient to reduce reproductive organ mass, whereas effects on sperm production were only detectable after two generations of inbreeding. We analyzed gene expression of four target genes, in order to find potential mechanisms of how inbreeding might affect sperm traits, and we expected a correlation between gene expression and sperm quality, since these genes have been reported to be relevant for normal sperm morphology or motility and related to fertility [61–64]. Contrary to our expectations, and even though inbreeding affected sperm traits, gene expression from the testes was not affected by our experimental breeding treatment. We also found no consistent correlations between gene expression and sperm quantity or quality.

Taken together, these results show that inbreeding altered male courtship behavior and courtship USVs emission, and negatively impacted male reproductive organs and sperm production. It is unclear whether these changes are sufficient to reduce male mating success, but they are consistent with evidence that inbreeding reduces male reproductive success [51] and may help to explain such findings. Our results confirm that our manipulation of genetic quality, i.e., experimental inbreeding, influenced the expression of male primary and secondary sexual traits.

### Correlations between pre- versus post-copulatory traits

We tested whether the emission of male courtship USVs (call number, duration or frequency) correlated with fertility traits, either positively or negatively, and if so, whether any such correlations depended upon our experimental inbreeding (Fig. 1). We recently found that USV length and number of simple syllables are negatively correlated with latency to reproduce [29], and that the numbers of short and ultra-high syllables emitted during the introduction phase are associated with mating (copulatory) success [26]. We were particularly interested in whether these vocal features provide a reliable index of fertility, and if so (and if females use them to assess males) then this could potentially explain why they correlated with mating and reproductive success. Contrary to our expectations (Condition-Dependent Fertility-Indicator or Tradeoff Hypothesis) though, we did not find any correlations between USV numbers and sperm concentration overall or within inbred or outbred males. However, during male–female interactions we found three parameters of USV emission (i.e., USV count, syllable length and mean frequency) that were negatively correlated with reproductive organ mass (relative testes and epididymal mass) regardless of our experimental breeding treatment, suggesting that there might be an overall tradeoff between these traits. Also, we found a negative correlation between the mean frequency of male USVs (during the introduction phase) and sperm concentration in 1^st^ generation inbred mice, but not other males.

### Limitations

There are a few limitations of our study that should be considered. First, we assumed that tradeoffs between investing in the development of primary versus secondary traits are long-lasting because (1.) investment into sperm production requires many days (spermatogenesis takes approximately 35 days in mice [65]), and (2.) individual male USV emission is stable for many parameters over at least three weeks [23]. However, some USV parameters depend upon several factors, including males'sexual arousal and health [16, 30], and also by the stimulus female’s estrous stage and behavior [12, 66, 67]. Thus, the longer time delay (72 ± 3 d) between recording USVs and sperm sampling in our study potentially explains the lack of consistent correlations between pre- and postcopulatory traits. Due to the invasive method of sperm collection in mice, repeated measurements of sperm parameters over time (to measure individual consistency) are not possible with current methods, and therefore, future studies should record USV emission at different timepoints within 35 days, including immediately before sperm sampling. Second, we manipulated male quality with experimental inbreeding, however, the mice in our study were kept under standard laboratory conditions with standardized access to resources, and tradeoffs between competing traits and negative effects of inbreeding can be more pronounced or only become apparent in competitive conditions [50, 51], under dietary restrictions [68], or other challenging environments [41]. Therefore, in addition to manipulating male quality, future studies on these questions could be conducted under more challenging environmental conditions. Third, it has been suggested that the direction of the relationship between pre-and post-copulatory traits can be positive or negative depending on several factors, such as genetic or environmental variation within and between individuals [3, 69]. Thus, individual variation can result in mixed evidence on pre- and post-copulatory tradeoffs and might be an explanation for some inconclusive results in our correlations between USVs, behavior, sperm production and reproductive organs.

## Conclusions

To our knowledge, this is the first study to test the effects of an experimental inbreeding on pre- or post-copulatory traits in house mice, or to study tradeoffs between these traits by manipulating male genetic quality. We found that inbreeding negatively impacted some pre-copulatory traits, including USV repertoire composition and courtship behavior. Inbreeding affected features of USVs but not call number, and our results are consistent with studies on other species [48, 49]. Inbreeding also negatively impacted post-copulatory traits, including both sperm quantity and quality, at least in the highly inbred males, as expected. Although, we did not find consistent correlations between USV and sperm quantity or quality, USV count and spectro-temporal parameters (syllable length and frequency) were negatively correlated with reproductive organ mass. Thus, we showed that pre- and postcopulatory traits in mice are both condition-dependent, but we detected little evidence for positive (Fertility-Indicator Hypothesis) or negative (Sexual Allocation Tradeoff Hypotheses) correlations between pre- versus post-copulatory traits in low or highly quality males.

## Methods

### Subjects and housing

Male subjects were wild-derived house mice, F5 descendants of wild-caught mice (*Mus musculus musculus*), trapped at the Konrad Lorenz Institute of Ethology, Vienna, Austria (for details see [70]). Stimulus females (*n* = 63) used for priming and during recordings were F1 offspring of mice caught at nine different locations in and around Vienna, Austria. To breed mice, we paired individuals trapped at different locations to avoid potential inbreeding. After weaning at 21 d, mice were kept in mixed-sex groups (≤ 4 siblings per cage) for an additional 2 weeks. At 5 weeks (35 d) of age, mice were separated by sex, females were housed in sister-pairs whenever possible, and males were housed individually to prevent fighting. Mice were housed in standard Type IIL cages (36.5 × 20 × 14 cm cages, Tecniplast, Germany) under standard laboratory conditions (mean ± SD room temperature: 22 ± 2 °C) with a 12:12 h light-red light cycle (red lights on at 15:00). Housing cages were provided with bedding (ABEDD, Austria), nesting material (Nestlet, Ehret, Austria), a nest box (Tecniplast, Germany) and a cardboard roll for enrichment. Food (rodent diet 1324, Altromin, Germany) and water were provided ad libitum and additionally 8 mg of a seed mixture were distributed in the bedding once every 2 weeks as additional dietary enrichment. Male subjects were recorded at 233 ± 16 d of age.

### Experimental breeding treatments

We investigated 63 male subjects (F5 generation) that were the offspring of 3 different experimental breeding treatments (*n* = 21 per group). 1^st^ generation inbred males (I) were offspring of experimental brother-sister matings (parents were siblings and grandparents were unrelated), 2^nd^ generation inbred males (II) were offspring of two generations of experimental brother-sister matings (parents and grandparents were siblings), and control males were outbred (O) (parents were 3^rd^ degree cousins and grandparents were unrelated). Stimulus females were all outbred, unfamiliar and unrelated to the males.

### USV recording

We used females in proestrus or estrus (“sexually receptive”) as stimuli for priming and recording males. Estrous states were assessed by vaginal smears in the morning of each recording day (between 10:00–12:00), and cell types were examined under a light microscope. Receptive females were identified by the absence of leukocytes in the smears by two independent observers [71]. Priming and recordings were conducted between 15:00–18:00 under red light within the active period of the mice. Wild mice often do not vocalize when sexually naïve, though brief exposure to a female 1 d before recording increases subsequent male USV emission for a female stimulus [16]. Therefore, all males were directly exposed to an unfamiliar stimulus female for 10 min within the males’ home cages 1 d before recording (“priming”).

Recordings (20 min) were conducted in a separate room, in a recording cage with bedding (modified Type III cage, Tecniplast, Germany; floor measurements: 36.5 × 21 × 15 cm, top measurements: 42.5 × 27 × 15 cm) and consisted of two 10 min phases (as described in Nicolakis, Marconi [29]). During the first phase (“introduction phase”, 10 min), the recording cage was divided into 2 compartments by a perforated plexiglass divider, which allowed the pair to have visual and olfactory stimuli. During this phase, the male compartment was covered with a metal grid and the stimulus female compartment was covered with plexiglass to minimize the recording of sounds produced in the female compartment [70]. Then, the plexiglass cover and the divider were manually removed to allow direct interactions between males and females and the pair was recorded for additional 10 min (“interaction phase”). Audio recordings were made with an ultrasound microphone (USG Electret Ultrasound Microphone, Avisoft Bioacoustics/Knowles FG) connected to a preamplifier (UltraSoundGate 416Hb, Avisoft Bioacoustics), and using recording software (RECORDER USGH software, Avisoft-RECORDER Version 4.2) with a sampling rate of 300 kHz and 16 bit format. Mice were video-recorded during both phases using an IP-camera (D-Link DCS-3710) and open-source software (iSpy—Video Surveillance Software) to analyze courtship behavior.

Stimulus females were used twice: first for USV recordings as stimulus for one male and then for priming another male to be recorded on the following day (Additional file 1: Fig. S1). Recordings were conducted over 3 weeks, on 4 consecutive days per week and 6 males per day (though only 3 males on the last recording day). On the first day of each week (day 0 in Additional file 1: Fig. S1) no experimental recordings were conducted, and, to also standardize the priming status of females, we exposed them to additional non-experimental males (using the same procedure as during experimental recordings), as female interactions with males during recordings might influence their subsequent behavior during the priming procedure. Thus, all females used for priming had similar socio-sexual experiences. This procedure was conducted three times (one time for each week), using a total of 18 additional non-experimental males. Recording order of the 3 breeding treatment groups was systematically balanced within and between recording days (i.e., 2 males/group/day for *n* = 6 males/day).

### USV analyses

Recordings were analyzed using STx software (Acoustic Research Institute, Vienna) and the Automatic Mouse USV Detector (A-MUD Version 3.2) [72] to detect USVs in sound files [16, 70]. We then visually checked the spectrograms and manually corrected false detections (false positives or negatives) and adjusted the length of detected segments when necessary. Ambiguous sounds were verified by acoustic inspection (slowed down 15–40 times). Visual inspection of spectrograms was conducted in STx with frequencies between 0–150 kHz displayed in a Hanning window with a range of 50 dB (floor at −80 dB), a frame of 4 ms and an overlap of 75%. Most studies on mice focus on USVs > 20 kHz, and yet mice also emit similar vocalizations at lower frequencies [14, 16]. Since sonic vocalizations are rarely studied in mice, and to keep USV analyses (especially mean frequencies) comparable with other studies, we classified calls as either ultrasonic vocalizations (> 20 kHz, USVs), or as sonic vocalizations (< 20 kHz, audible to humans). Sonic vocalizations were classified as either "mid-frequency vocalizations" (MFVs) or “squeaks”. MFVs are single tone sounds between 15 to 20 kHz with a short duration and sometimes contain 1 harmonic element [16, 26], and have been reported to be emitted under distress, such as isolation and restraint [14], but also during courtship [16]. Squeaks are broadband vocalizations (also called BBVs) with multiple harmonic elements and F0 < 20 kHz that are often emitted during agonistic interactions or female defensive behavior [57, 58], but also during mating, when female BBVs are synchronized with males USVs [26]. Ultrasonic vocalizations (USVs) are whistle-like, pure-tone calls emitted entirely or partially > 20 kHz (mainly between 50–90 kHz) and have been classified into different syllable types according to their spectro-temporal shape (i.e., duration and frequency modulation), as previously described [29]. We originally manually classified USVs emitted during the introduction phase into 15 different USV types as previously reported. However, as manual classification is very time-consuming [73], we also simplified our classification by using 10 syllable types in the interaction phase by merging long USVs without frequency jumps (i.e., “f”, “up”, “d”, “u”, “ui”, “c”) into one category of “simple” USVs (see Additional file 1: Table S1). We only used this simpler classification for the interaction phase. Still, we also provide additional analyses of the repertoire composition during the introduction phase using all 15 USV types (Additional file 1), to facilitate comparison with previous studies, which used 15 types [16, 23, 29]. Additionally, we conducted analyses on 3 USV categories consisting of the most frequently used syllable types (“short”: all USVs < 10 ms, “simple”: all USVs consisting of 1 element, and “complex”: all USVs consisting of > 1 element; see Additional file 1: Table S1), to facilitate statistical analyses. We run A-MUD to compute and extract spectro-temporal parameters for each vocalization, and all parameters were exported and used for statistical analyses. Count parameters included the *total number of vocalizations* (total count of all USVs, MFVs or squeaks, respectively, for each 10 min recording), the *vocal repertoire* (number of different syllable types detected in each 10 min recording) and the *repertoire composition* (number of USVs of each syllable type). Spectro-temporal parameters included *USV length* (duration in ms of each USV), *mean frequency* (mean frequency of each USV in kHz), *intersyllable interval* (ISI, duration between two syllables within one bout, i.e., within one sequence of USVs with intervals < 300 ms) and *interbout interval* (IBI, duration between bouts, i.e., intervals between syllables of > 300 ms) [23]. We excluded unclassifiable (i.e., unstructured) USVs for the calculation of the spectro-temporal parameters, because their boundaries (start & end, min. & max. frequency) are difficult to define due to their broadband and unstructured shape [14, 29, 74].

### Behavioral analyses

Courtship behavior was analyzed using video recordings from both phases. Analyses were conducted manually by an observer blind to the breeding treatments using NOLDUS Observer XT 7.0 software (Noldus Information Technology, The Netherlands). During the introduction phase, we analyzed male behavior, and we used the following parameters for further analyses: the number of times the male investigated the divider and the total time spent investigating it (while the female was not close to the divider) and the number and duration of male–female interactions through the divider. During the interaction phase we analyzed male and female behaviors with the following variables: number of female defensive behaviors (the female stretching out forelimbs up to the point until they are clearly visible in front of her body and preparing to or actually pushing away the male); and number and duration of males' female-directed investigatory behavior (male investigating the female, either by direct anogenital sniffing or body sniffing, social grooming or also by indirect investigation like sniffing in the direction of the female with an obvious indication to gather olfactory information from her and/or following in the female path exactly and trying to get (closer) to her). No mating or copulation attempts were observed during direct interactions.

### Sperm collection and analyses

Sperm from each male were collected at the end of the experiment, 72 ± 3 d after USV recordings (*n* = 62). One male (I) died between USV recording and sperm analysis, and thus sample sizes were: *n* = 21 (O), *n* = 20 (I), *n* = 21 (II) males. Males were sacrificed by an overdose of CO_2_ and we measured body mass (g), body length (cm, from the snout to the base of the tail) and anogenital distance (cm). We then dissected both testes, caudae epididymides and seminal vesicles. We weighed the organs with a scale (Ohaus PIONEER Analytical Balance PX124) and transferred both caudae epididymides into a pre-warmed (37 °C) 35 mm culture dish containing a 1 ml drop of THY medium covered with mineral oil. We cut the epididymides 3–5 times and allowed the spermatozoa to swim out for 10 min at 37 °C. Afterwards, we removed the tissue and homogenized the sperm suspension by carefully rotating the dish for 1 min before performing computer assisted sperm analysis (CASA). The CASA system (SCA®, Sperm Class Analyzer, Microptics, Spain) consisted of a high-resolution camera (Basler acA1300-200uc, Basler, Germany) connected to a phase-contrast microscope (Nikon Eclipse E200, Nikon, Japan) using a 10 × objective (Nikon 10 x/0,25 Ph1 BM, Nikon, Japan) under negative phase contrast to track and record individual sperm. We loaded 3 µl of the homogenized sperm suspension on a pre-warmed (37 °C) Leja® slide (20 µm deep; Leja Products BV, Netherlands), before five randomly selected microscopic fields were captured (frame rate 50 fps; 25 images per field) at evenly distributed sites throughout the slide to ensure an objective sample estimate. We used the SCA® software (Version 6.2.0.0.) to determine sperm concentration (million/ml), sperm motility (percentage of sperm moving faster than 10 µm/s), sperm curvilinear velocity (VCL in µm/s, velocity along the actual sperm path), straight-line velocity (VSL in µm/s, velocity along a straight line between the first and last position of a sperm track) and average path velocity (VAP in µm/s, time-averaged velocity of a sperm head along its average path). Blinded observers inspected all videos and performed manual corrections on sperm tracks when necessary (Additional file 1: Fig. S2). Sperm analysis was performed twice, once immediately after sperm collection (Timepoint 0: T0), and then 2 h after the first measurement (Timepoint 2: T2). We used the second measurement to calculate the reduction in sperm motility and swimming velocities over time to estimate sperm longevity.

### Gene expression

We aimed to analyze gene expression levels of four target genes which are associated with spermatogenesis: *Protamine-2*, *GAPDS* (glyceraldehyde-3-phosphate dehydrogenase, spermatogenic), *STK22B* (testis-specific serine kinase substrate) *and Park-2 (*parkin RBR E3 ubiquitin protein ligase). All of these genes are protein coding genes expressed in mouse testes and are relevant for physiological development of sperm morphology or motility or for regulations of energy pathways and sperm mitochondria, respectively [61–64] (for more details see Additional file 1).

For gene expression analysis, dissected testes were stored in RNAlater (Invitrogen, AM7021) at 4 °C for at least 24 h and subsequently stored at −80 °C until RNA extraction. RNA was extracted using the RNeasy Mini Kit (Qiagen, Hilden, Germany) according to the manufacturer’s instructions. After elution DNAseI-treatment (DNAse I Set, Zymo Research, E1010) was conducted to eliminate DNA contamination from the RNA which was then reverse-transcribed to synthesize complementary DNA (cDNA) using Applied Biosystems High-Capacity cDNA Reverse Transcription Kit, following the manufacturer’s instructions. Gene expression levels were then analyzed from cDNA using Droplet Digital PCR (ddPCR™). Expression levels were estimated using probe-based assays on a QX200™ Droplet Reader (Bio-Rad) and analyzed with the Bio-Rad Droplet Digital™ PCR QuantaSoft software. The reference gene *RPL38* (Ribosomal Protein L38) was used to calculate relative concentrations (copies/µl) of the target genes (*Protamine-2*, *GAPDS*, *Park-2* and *STK22B*) A list of used sequences for primers and probes is shown in Additional file 1: Table S2. Additional details on gene expression methods can be found in Additional file 1: Table S3.

### Statistical analyses

Data distributions and homogeneity of variances were examined separately for each recording phase or sperm sampling timepoint (Kolmogorov–Smirnov and Levene’s test). We used transformed data whenever necessary; however, USV count data were still extremely right-skewed, and could not be normalized. Results are presented as mean ± SD, unless stated otherwise, and two-tailed tests were used and considered significant at α ≤ 0.05. Statistical tests were conducted using SPSS (IBM SPSS Statistics 27) and RStudio (R-Version 3.5.1 [75]).

Vocalizations were analyzed using generalized linear mixed models (GZLMM, conducted in SPSS), with breeding treatment, recording phase and the interaction term treatment*phase as fixed effects and mouse ID as random effects to control for repeated measures. For count data of vocalizations (number of USVs, MFVs, squeaks and vocal repertoire) negative binomial models were conducted, due to the skewness of the data. Post-hoc pairwise comparisons were conducted using a sequential step-down Sidak method (Holm-Sidak method) to correct for multiple testing. Residuals were checked for normal distribution using Kolmogorov–Smirnov test and visual inspections of histograms. We conducted additional Kruskal–Wallis tests to investigate inbreeding effects on the three most frequent USV categories (“short”, “simple” and “complex” types, see Additional file 1: Table S1).

To investigate the effects of inbreeding on USV spectro-temporal features, we conducted two analyses. First, we used average values calculated over all USVs for each individual and phase (i.e., means and grand means, *n* = 63 males, see [29]) using GZLMMs in SPSS as described above, but using linear models instead of negative binomial models. Second, we analyzed spectro-temporal parameters when using single datapoints extracted for each USV emitted during the interaction phase (*n* = 17 636 USVs). Here we used GZLMM models in R (package “lme4”) as described in [16], we calculated two models; a null model with an intercept only and a “breeding treatment” model, comparing the three breeding treatment groups, with mouse ID as random intercept. AICc (Akaike’s Information Criterion) was computed for each model to assess the best fitted model [16].

To compare the repertoire composition of different syllable types between the 3 breeding treatments, we conducted a multivariate and non-parametric analyses of similarities (ANOSIM) on the number of USVs emitted within each syllable type (using the functions “vegdist”, “anosim” and “metaMDS” in the package “vegan” [76]. The ANOSIM statistic compares the mean of ranked dissimilarities between groups with the mean of ranked dissimilarities within groups and was ran with 999 permutations and the Bray–Curtis dissimilarity as a distance measure based on ranks [16]. Results were visualized using nMDS (non-metric multidimensional scaling) based on the Bray–Curtis dissimilarity index, to represent similarities between groups in a 2-dimensional plot.

Sperm measurements were analyzed using linear GZLMMs (conducted in SPSS) with breeding treatment, timepoint and the interaction term treatment*timepoint as fixed effects and mouse ID as random effects to control for repeated measures. Post-hoc pairwise comparisons were conducted using a sequential step-down Sidak method (Holm-Sidak method) to correct for multiple testing. Residuals were checked for normal distribution using Kolmogorov–Smirnov test and visual inspections of histograms. Effects of inbreeding on male reproductive organs and gene expression were analyzed using ANOVA or Kruskal–Wallis tests for parametric and non-parametric variables, respectively.

To test for correlations between pre- and/or postcopulatory traits and between traits and behavior Spearman rank correlations were conducted in SPSS. To test whether the slopes of the correlations differed between the 3 breeding treatments, each variable was transformed to z-scores, general linear models (GLM) were conducted, and the interaction term of the model between variable and treatment group indicated significant differences in the slopes between treatment groups. For correlations between pre-and postcopulatory traits we first aimed to test for correlations between USV and sperm quantity (i.e., number of USVs and sperm concentration). Since both USV recordings and sperm analyses contain several different parameters, but studies on correlations between courtship vocalizations and sperm traits are rare, we conducted additional exploratory correlations to test for relationships between USV and sperm quality. Since these were exploratory analyses, we report results before and after FDR (false discovery rate) correction for multiple testing; future studies are needed to conduct confirmatory analysis.

## Supplementary Information

Additional file 1

## Data Availability

The datasets generated and/or analyzed during the current study are available in the repository of the University of Veterinary Medicine Vienna Phaidra. Dataset: Excel file including 2 data sheets and their respective legends and data description: (1) raw data including USV summary data, sperm measurements, measures of reproductive organs, gene expression levels, and behavioral data; and (2) table containing single datapoints for each USV recorded during the interaction phase. https://phaidra.vetmeduni.ac.at/o:3828 (77). R-codes: Text file including R-codes used to run (1) ANOSIM and nMDS plots on syllable type usage, and (2) GLMMs to test for treatment effects on spectro-temporal features using single USV datapoints. https://phaidra.vetmeduni.ac.at/o:3834 (78).

## Abbreviations

AIC: Akaike’s Information Criterion
A-MUD: Automatic Mouse USV Detector
ANOSIM: Analyses of similarities
BBV: Broadband vocalization
CASA: Computer assisted sperm analysis
FDR: False discovery rate
GAPDS: Glyceraldehyde-3-phosphate dehydrogenase, spermatogenic
GLM: General linear model
GZLMM: Generalized linear mixed model
IBI: Inter-bout interval
IQR: Interquartile range
ISI: Inter-syllable interval
MFV: Mid-frequency vocalization
NMDS: Non-metric multidimensional scaling
Park-2: Parkin RBR E3 ubiquitin protein ligase
STK22B: Testis-specific serine kinase substrate
USV: Ultrasonic vocalization
VAP: Average path velocity
VCL: Curvilinear velocity
VSL: Straight linear velocity
