# Supplementary material for: Pre- and post-copulatory traits are affected by experimental inbreeding, but they are not correlated
Source: BMC Biol. 2025 May 28;23:144. doi: 10.1186/s12915-025-02245-4 (PMC12121127; doi:10.1186/s12915-025-02245-4)
Supplement: Supplementary file 1 — Additional file 1 [file 12915_2025_2245_MOESM1_ESM.pdf]

Supplementary Material

1 Supplementary Methods

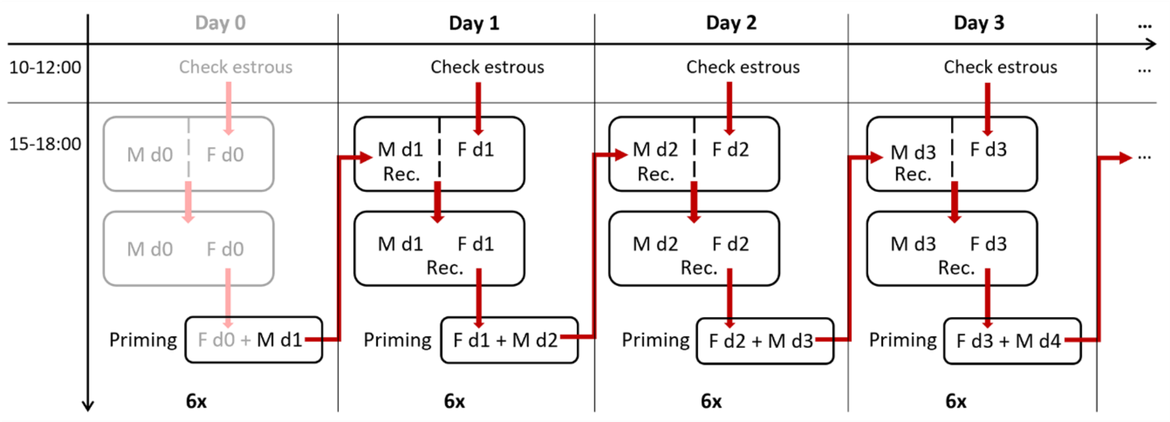

Supplementary Figure 1. Procedure for male sexual priming and recording. To standardize and only choose sexually receptive females (F) (proestrus or estrus) as male stimuli, their estrous state was first determined during the white light phase (10:00-12:00). Then, during the start of the red-light phase (15:00-18:00), males (M) were recorded (Rec.) with the presence of a stimulus F, first while separated by a perforated divider (10 min), then during direct interactions (10 min). The same stimulus F was also used to prime another male for the next recording day (d1-d4) (experimental animals shown in black font). Priming F used for the first group of M (F d0), were previously exposed to additional non-experimental M (M d0) using the same protocol to ensure similar socio-sexual experience (Day 0) (shown in grey font). Arrows show the timeline of the procedures for each animal.

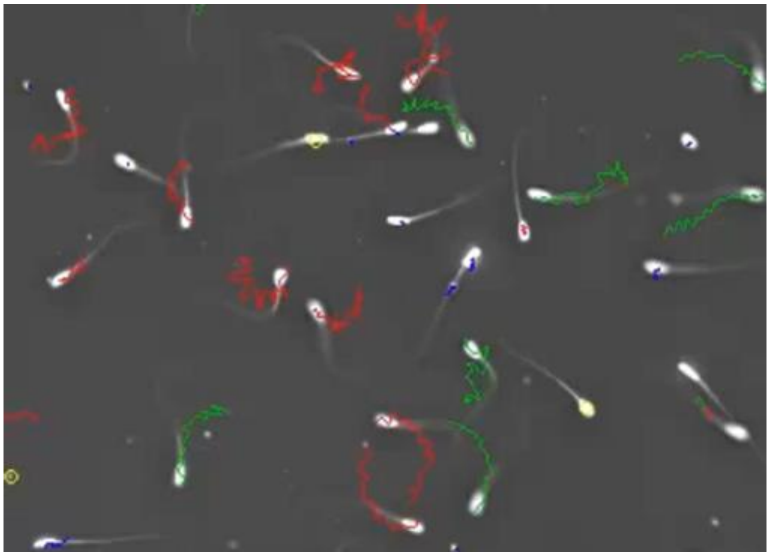

Parameter	Setting
Area (min-max)	23-199 µm²
Drifting	0 µm/s
Static	<10 µm/s
Progressive	STR > 50
VAP points	50 pixels
Connectivity	18 pixels

Supplementary Figure 2. Example of sperm trajectories visualized in the Sperm Class Analyzer (SCA®, Version 6.2.0.0., Microptics, Spain) of the CASA system (computer assisted sperm analysis, CASA) and the used settings for sperm analysis.

Supplementary Table 1. Different USV classifications that we used: from 15 to 5 types (adapted from Nicolakis et al. (1)). We used 15 types in the introduction phase to be able to compare our results with previous studies. However, we also used simpler classifications (10 types for the introduction and interaction phase) and we also pooled syllable types for some analyses.

15 USV Types (introduction phase)		10 USV types (interaction phase)		Pooled syllable types
**USV label**	**USV shape**	**USV label**	**USV shape**	
us	ultra short (< 5 ms)	us	ultra short (< 5 ms)	short
s	short (< 10 ms)	s	short (< 10 ms)	short
f	flat (< 5 kHz frequency modulation)	simple	USV consisting of 1 element	simple
up	up (> 5 kHz increase)	simple	USV consisting of 1 element	simple
d	down (> 5 kHz decrease)	simple	USV consisting of 1 element	simple
u	u-shaped (> 5 kHz modulation)	simple	USV consisting of 1 element	simple
ui	u-shaped inverted (> 5 kHz modulation)	simple	USV consisting of 1 element	simple
c	complex (≥ 2 directional changes (> 5 kHz)	simple	USV consisting of 1 element	simple
c2	USV with 1 frequency jump	c2	USV with 1 frequency jump	complex
c3	USV with 2 frequency jumps	c3	USV with 2 frequency jumps	complex
c4	USV with 3 frequency jumps	c4	USV with 3 frequency jumps	complex
c5	USV with ≥ 4 frequency jumps	c5	USV with ≥ 4 frequency jumps	complex
h	harmonic (USV with harmonic element)	h	USV with harmonic element	complex
uh	ultra high (> 91 kHz)	uh	ultra high (> 91 kHz)	ultra high
uc	unclassifiable (unstructured shape)	uc	unclassifiable (unstructured shape)	unclassifiable

Target genes for spermatogenesis:

Protamine 2 is a DNA binding protein, that enables sperm chromatin condensation into a small volume, and thus leads to normal sperm head morphology. Deficiency in Protamine-2 leads to sperm DNA damage and can impact sperm function and male fertility (reviewed in (2)). *GAPDS* encodes a sperm-specific enzyme during spermatogenesis that regulates energy-producing pathways required for sperm motility. It is bound to the fibrous sheath of the principal piece of the sperm flagellum, and thus involved in flagellated sperm motility and male fertility (3). *STK22B* encodes testis-specific serine/threonine kinases which have been found in the cytoplasm of elongating spermatids and accumulated around the base of the flagellum. Loss of these enzymes is associated with abnormal spermatogenesis and infertility (4). *Park-2* enables ubiquitin protein ligase activity which induces selective autophagy of damaged mitochondria. Sperm mitochondria are necessary for energy production and thus normal sperm motility; however, the transmission of paternal mitochondria can have deleterious effects. Ubiquitination of sperm mitochondria during spermatogenesis facilitate the elimination of paternal mitochondria after fertilization, where specific autophagy-related ubiquitin-binding proteins promote autophagy of ubiquitinated mitochondria (reviewed in (5)).

Supplementary Table 2. Sequences for primers and probes used for four target genes (Protamine-2, Park2, STK22B, GAPDS) and one reference gene (RPL38).

**Gene**	**official symbol and NCBI-Gene ID**	**Probe sequence (5' → 3'), modifications for all: 5' FAM, 3' BHQ-1**
Protamine-2	Prm2, 14447	TGACTTCCTTGGCTCCAGGCAGAAT
Park-2	Prkn, 50873	AGGGCTTCACAGTCAAAGCCA TGA
STK22B	Tssk2, 22115	TCT ACA TTG TCA TGG AGC TGGGCG
GAPDS	Gapdhs, 14447	CCCAGTGGACGATGGGATGATGTT
RPL38	Rpl38, 67671	CCATGCCTCGGAAAATTGAGGAGATC
**Gene**	**Primer sequence (5' → 3')**	
Protamine-2	forward	CCACAAGAGGCGTCGGTCAT
	reverse	GGCAGGTGACTATTCCTTGGG
Park-2	forward	CACAACAGACGTTCCAGGTGC
	reverse	CTCAAGGGCGATTGGGAAG
STK22B	forward	GACCGTCAACCACCGTTCCA
	reverse	GGGCTCCTCGGCACTTGATG
GAPDS	forward	GGATGGGCCATCAAAGAAGG
	reverse	CGGCAGGTCAGGTCCACAAC
RPL38	forward	CGTTCTCTTCGGTTCTCATCGC
	reverse	CTTCCGCCGGGCTGTCAG

Supplementary Table 3: Information on gene expression Methods based on dMIQE2020 checklist for authors (see Huggett et al. (6)).

ITEM TO CHECK	PROVIDED	COMMENT
	**Y/N**	
**1. SPECIMEN**		
Detailed description of specimen type and numbers	**Y**	see Methods section "Gene expression": Testes from 62 male mice; stored in RNAlater (Invitrogen, AM7021) at 4°C for at least 24h and subsequently stored at -80°C until RNA extraction.
Sampling procedure (including time to storage)	**Y**	
Sample aliquotation, storage conditions and duration	**Y**	
**2. NUCLEIC ACID EXTRACTION**		
Description of extraction method including amount of sample processed	**Y**	see Methods section "Gene expression": RNA was extracted using the RNeasy Mini Kit (Qiagen, Hilden, Germany) according to the manufacturer's instructions. (Required details are reported in the manufacturers instructions: https://www.qiagen.com/us/resources/resourcedetail?id=f646813a-efbb-4672-9ae3-e665b3045b2b&lang=en)
Volume of solvent used to elute/resuspend extract	**Y**	
Number of extraction replicates	**Y**	
Extraction blanks included?	**Y**	
**3. NUCLEIC ACID ASSESSMENT AND STORAGE**		
Method to evaluate quality of nucleic acids	**Y**	NanoDrop
Method to evaluate quantity of nucleic acids (including molecular weight and calculations when using mass)	**Y**	NanoDrop
Storage conditions: temperature, concentration, duration, buffer, aliquots	**Y**	-80°C in TE buffer solution
Clear description of dilution steps used to prepare working DNA solution	**Y**	After elution DNAseI-treatment (DNAse I Set, Zymo Research, E1010) (detailed information can be found here: https://files.zymoresearch.com/datasheets/_e1010_dnase_i_and_dna_digestion_buffer_set.pdf)
**4. NUCLEIC ACID MODIFICATION**	**NA**	N/A
Template modification (digestion, sonication, pre-amplification, bisulphite etc.)	**N**	N/A
Details of repurification following modification if performed	**N**	N/A
**5. REVERSE TRANSCRIPTION**	**Y**	see Methods section "Gene expression": RNA which was then reverse-transcribed to synthesize complementary DNA (cDNA) using Applied Biosystems High-Capacity cDNA Reverse Transcription Kit, following the manufacturer's instructions, and stored at -20°C until further processing. (Required details are reported in the manufacturer's instructions: https://assets.thermofisher.com/TFS-Assets/LSG/manuals/MAN0017977_highcap_cDNA_RT_UG.pdf)
cDNA priming method and concentration	**Y**	
One or two step protocol (include reaction details for two step)	**Y**	
Amount of RNA added per reaction	**Y**	
Detailed reaction components and conditions	**Y**	
Estimated copies measured with and without addition of RT*	**Y**	
Manufacturer of reagents used with catalogue and lot numbers	**Y**	
Storage of cDNA: temperature, concentration, duration, buffer and aliquots	**Y**	
**6. dPCR OLIGONUCLEOTIDES DESIGN AND TARGET INFORMATION**		
Sequence accession number or official gene symbol	**Y**	see Supplementary Table 2
Method (software) used for design and *in silico* verification	**Y**	
Location of amplicon	**Y**	
Amplicon length	**Y**	
Primer and probe sequences (or amplicon context sequence)**	**Y**	

Location and identity of any modifications	**Y**	
Manufacturer of oligonucleotides	**Y**	
**7. dPCR PROTOCOL**		
Manufacturer of dPCR instrument and instrument model	**Y**	see Methods section "Gene expression": Gene expression levels were then analyzed from cDNA using Droplet Digital PCR (ddPCR™). Expression levels were estimated using probe-based assays on a QX200™ Droplet Reader (Bio-Rad) and analyzed with the Bio-Rad Droplet Digital™ PCR QuantaSoft software according to instructions.
Buffer/kit manufacturer with catalogue and lot number	**Y**	
Primer and probe concentration	**Y**	
Pre-reaction volume and composition (incl. amount of template and if restriction enzyme added)	**Y**	
Template treatment (initial heating or chemical denaturation)	**Y**	
Polymerase identity and concentration, Mg++ and dNTP concentrations***	**Y**	
Complete thermocycling parameters		
**8. ASSAY VALIDATION**		
Details of optimisation performed	**Y**	Analyses were run in replicates and using positive and negative controls
Analytical specificity (vs. related sequences) and limit of blank (LOB)	**N**	N/A
Analytical sensitivity/LoD and how this was evaluated	**N**	N/A
Testing for inhibitors (from biological matrix/extraction)	**N**	N/A
**9. DATA ANALYSIS**		
Description of dPCR experimental design	**Y**	Each sample was run in duplicates; positive and negative values were analyzed all together with the samples on the QuantaSoft Software. The reference gene RPL38 (Ribosomal Protein L38) was used to calculate relative concentrations (copies/ µl) of the target genes. Expression levels are given as the relative ratio of the concentration (copies·µl-1) of the assay target gene over the concentration of the reference gene. Primer pairs were first tested on high quality cDNA extracts to verify amplification of the target gene. Effects of inbreeding were analyzed using ANOVA or Kruskal-Wallis tests for parametric and non-parametric data, respectively. Sperman correlations were conducted to test for correlations with sperm production.
Comprehensive details negative and positive of controls (whether applied for QC or for estimation of error)	**Y**	
Partition classification method (thresholding)	**Y**	
Examples of positive and negative experimental results (including fluorescence plots in supplemental material)	**N**	
Description of technical replication	**Y**	
Repeatability (intra-experiment variation)	**Y**	
Reproducibility (inter-experiment/user/lab etc. variation)	**Y**	
Number of partitions measured (average and standard deviation)	**N**	
Partition volume	**N**	
Copies per partition (λ or equivalent) (average and standard deviation)	**N**	
dPCR analysis program (source, version)	**Y**	
Description of normalisation method	**Y**	
Statistical methods used for analysis	**Y**	
Data transparency	**Y**	

* Assessing the absence of DNA using a no RT assay (or where RT has been inactivated) is essential when first extracting RNA. Once the sample has been validated as DNA-free, inclusion of a no-RT control is desirable, but no longer essential.

** Disclosure of the primer and probe sequence is highly desirable and strongly encouraged. However, since not all commercial pre-designed assay vendors provide this information when it is not available assay context sequences must be submitted (Bustin et al. Primer sequence disclosure: A clarification of the miqe guidelines. Clin Chem 2011;57:919-21.)

*** Details of reaction components is highly desirable, however not always possible for commercial disclosure reasons. Inclusion of catalogue number is essential where component reagent details are not available.

2 Supplementary Results

2.1 Pre-copulatory traits

2.1.1 Ultrasonic vocalizations and courtship behavior

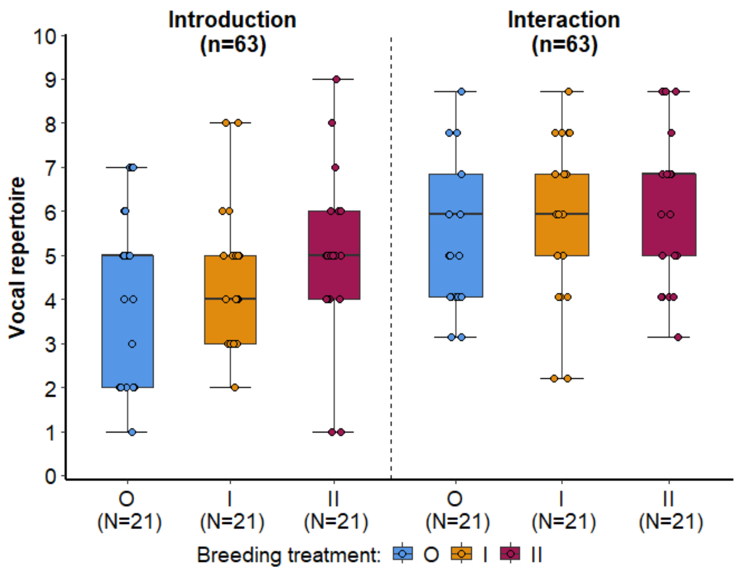

Supplementary Figure 3. Vocal repertoire size during the introduction and interaction phase, comparing 3 breeding treatments. Points represent raw data, boxplots show median (center line), interquartile range (IQR, box), and 1.5x IQR (whiskers) of the USV repertoire size, emitted by outbred (O, blue), 1st generation inbred (I, orange) and 2nd generation inbred (II, red) mice (shown from left to right, respectively).

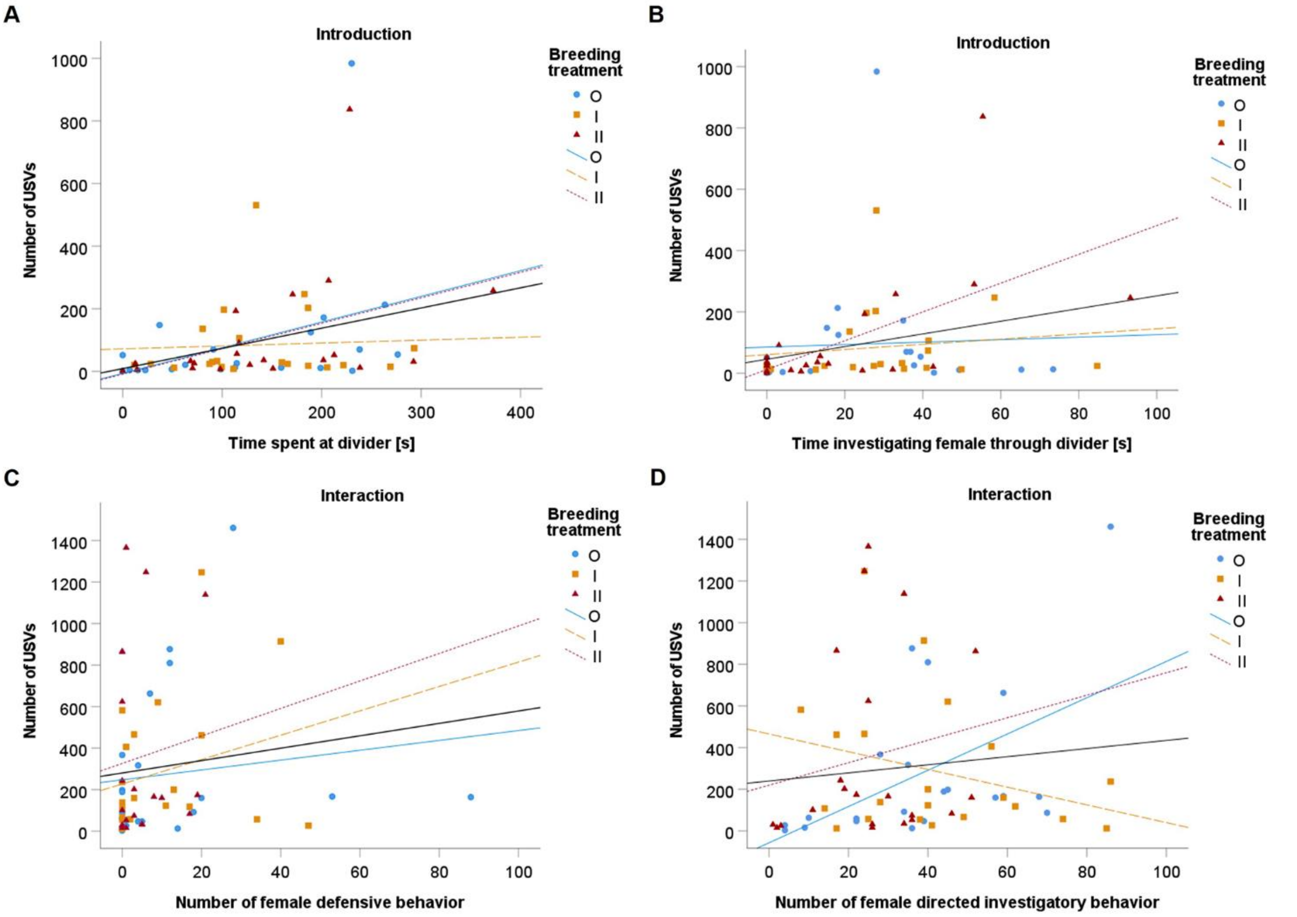

Supplementary Figure 4. Correlation between behavior and USV emission. Symbols represent outbred (O, blue circles, solid line), 1st generation inbred (I, orange squares, dashed line) and 2nd generation inbred (II, red triangles, dotted line) males. The black line represents the overall correlation. Correlations between number of USVs and (**A**) time spent at the divider, (**B**) time spent investigating the female through the divider, (**C**) number of female defensive behaviors, and (**D**) number of female-directed investigatory behavior during either (**A** and **B**) the introduction phase or (**C** and **D**) the interaction phase.

To analyze vocal repertoire composition, we also conducted additional analyses using all 15 syllable types during the introduction phase to be able to compare our data to previous studies. The USV repertoire composition of the introduction phase did not differ among the three breeding treatments (ANOSIM: Introduction: 15 syllable types, R=0.003, p= 0.378, Fig. S5C). Yet, when we pooled the two inbred treatments, the repertoire composition differed significantly from the outbred males (ANOSIM: introduction: 15 syllable types, R=0.089, p=0.045).

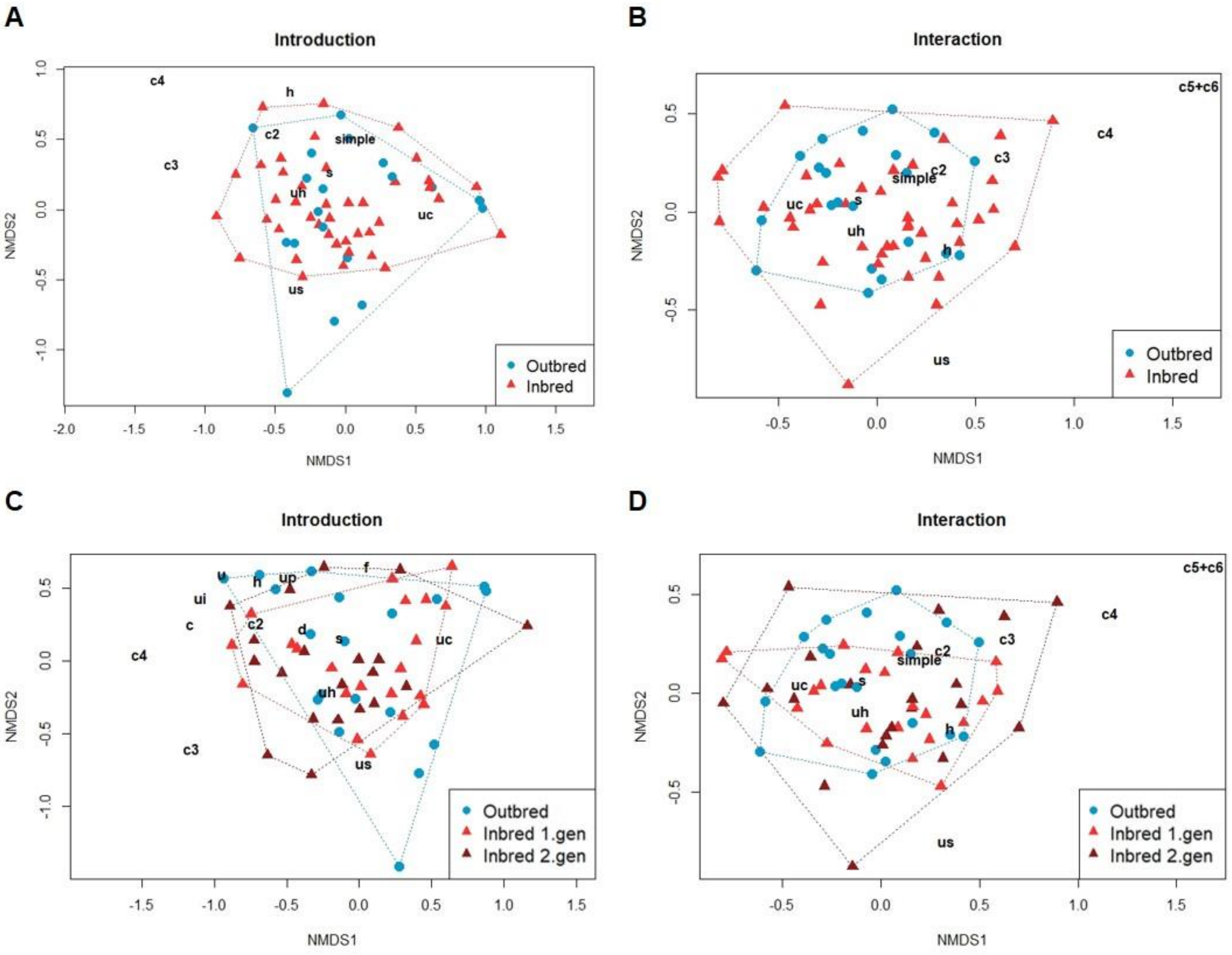

Supplementary Figure 5. Syllable type usage presented in non-metric multidimensional scaling (nMDS) plots. NMDS plots of syllable types emitted during the introduction (**A** and **C**) and interaction phase (**B** and **D**). (**A** and **B**) Plots compare outbred (blue dots) versus inbred (red triangles, pooled 1st and 2nd generation inbred mice) mice when using 10 syllable types (i.e., simple types pooled into one category, see Table S1). (**C** and **D**) Plots compare outbred (blue dots), 1st generation inbred (red triangles) and 2nd generation inbred (dark red triangles) mice, when using 15 syllable types during the introduction phase and 10 syllable types during the interaction phase. Letters in black indicate the syllable types and each symbol represents one recorded pair (i.e., outbred or inbred F5 male + outbred F1 female). Distances between the symbols represent similarities of pairs in the syllable type usage. Short distances of symbols to letters indicate syllable types that were most representative for each pair.

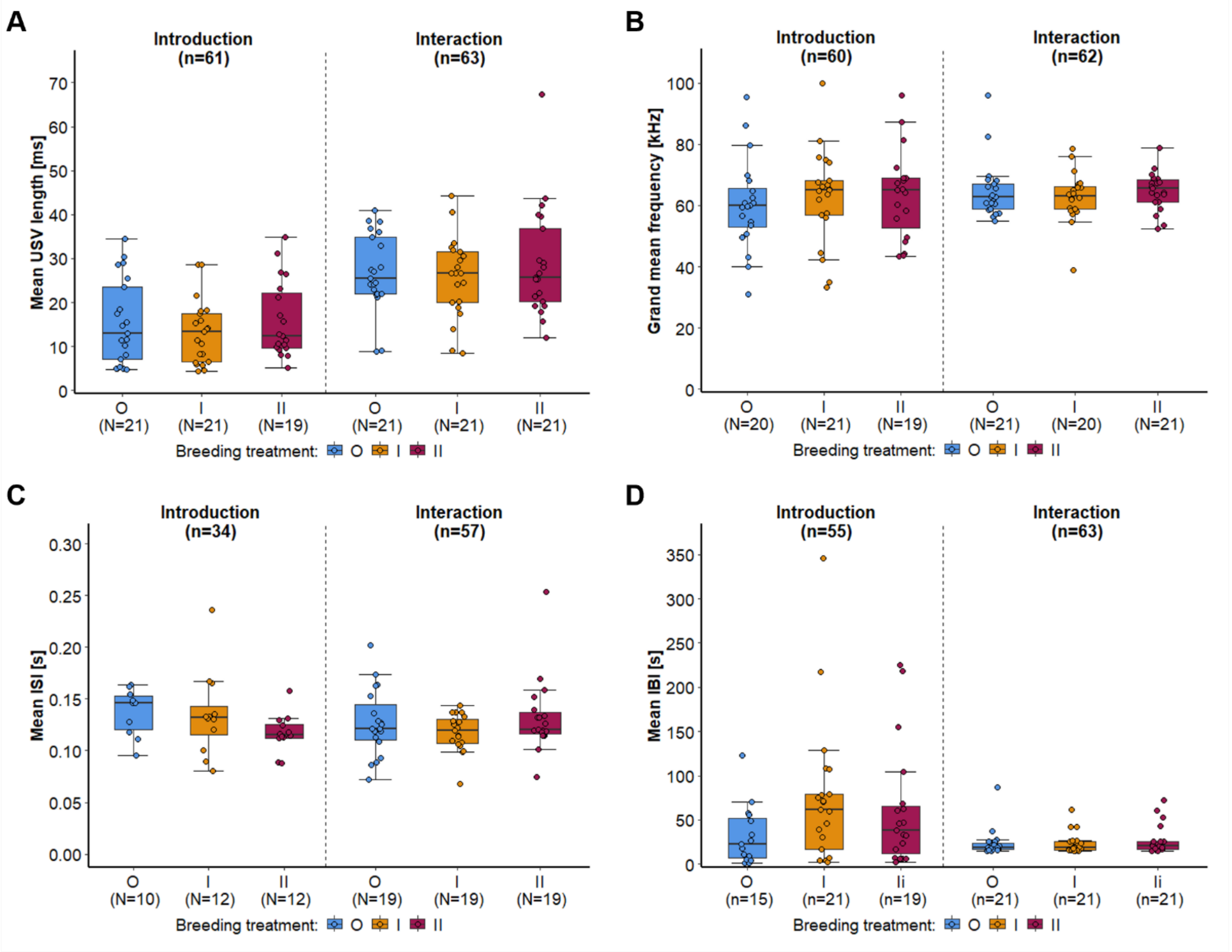

Supplementary Figure 6. Spectro-temporal features of USVs emitted during the introduction versus interaction phase comparing 3 breeding treatments. Points represent raw data, boxplots show median (center line), interquartile range (IQR, box), and 1.5x IQR (whiskers) of (**A**) mean USV length (ms), (**B**) grand mean frequency (kHz), (**C**) mean inter-syllable interval (ISI) and (**D**) mean inter-bout interval (IBI), of USVs emitted by outbred (O, blue), 1st generation inbred (I, orange) and 2nd generation inbred (II, red) mice (shown from left to right, respectively).

2.1.2 Sonic vocalizations and behavior

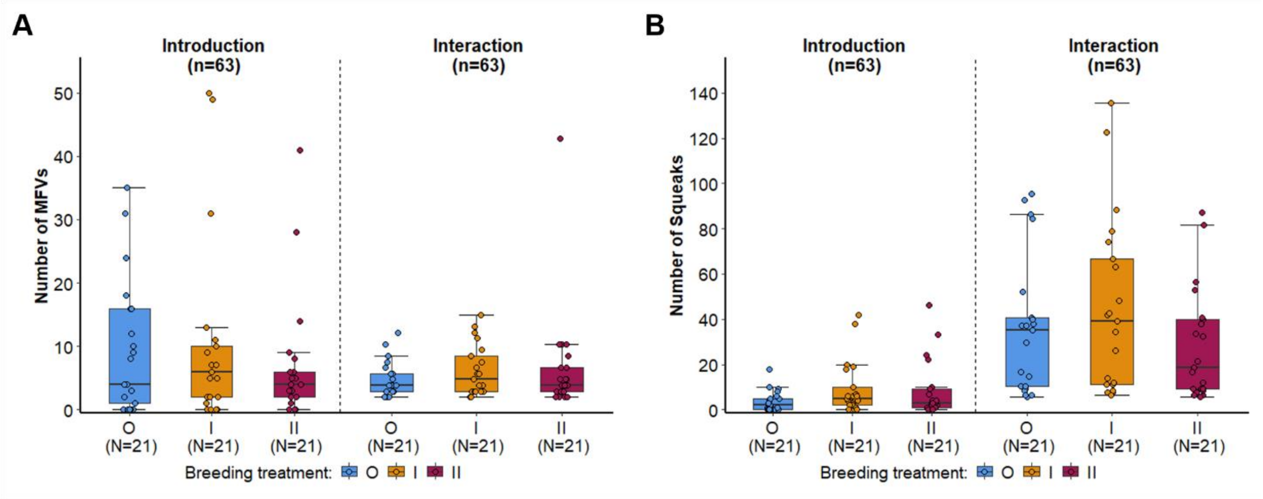

Supplementary Figure 7. Sonic vocalizations emitted during the introduction versus interaction phases comparing 3 breeding treatments. Points represent raw data, boxplots show median (center line), interquartile range (IQR, box), and 1.5x IQR (whiskers) of (**A**) number of MFVs and (**B**) number of squeaks, emitted by outbred (O, blue), 1st generation inbred (I, orange) and 2nd generation inbred (II, red) mice (shown from left to right, respectively).

2.1.3 Correlations between sonic and ultrasonic vocalizations

Exploratory statistics showed that the number of USVs was positively correlated with the number of squeaks during the introduction phase (rs=0.358, p=0.004), but not during direct interactions rs= 0.150, p=0.242). On the contrary, the number of USVs was positively correlated with the number of MFVs during direct interactions (rs= 0.288, p=0.022), but this correlation was not significant during the introduction phase (rs=0.210, p=0.098). The number of squeaks did not significantly correlate with the number of MFVs in either phase (introduction: rs=0.056, p=0.665, interaction: rs= 0.205, p=0.106).

2.2 Post-copulatory traits:

2.2.1 Gene expression & correlation with sperm traits:

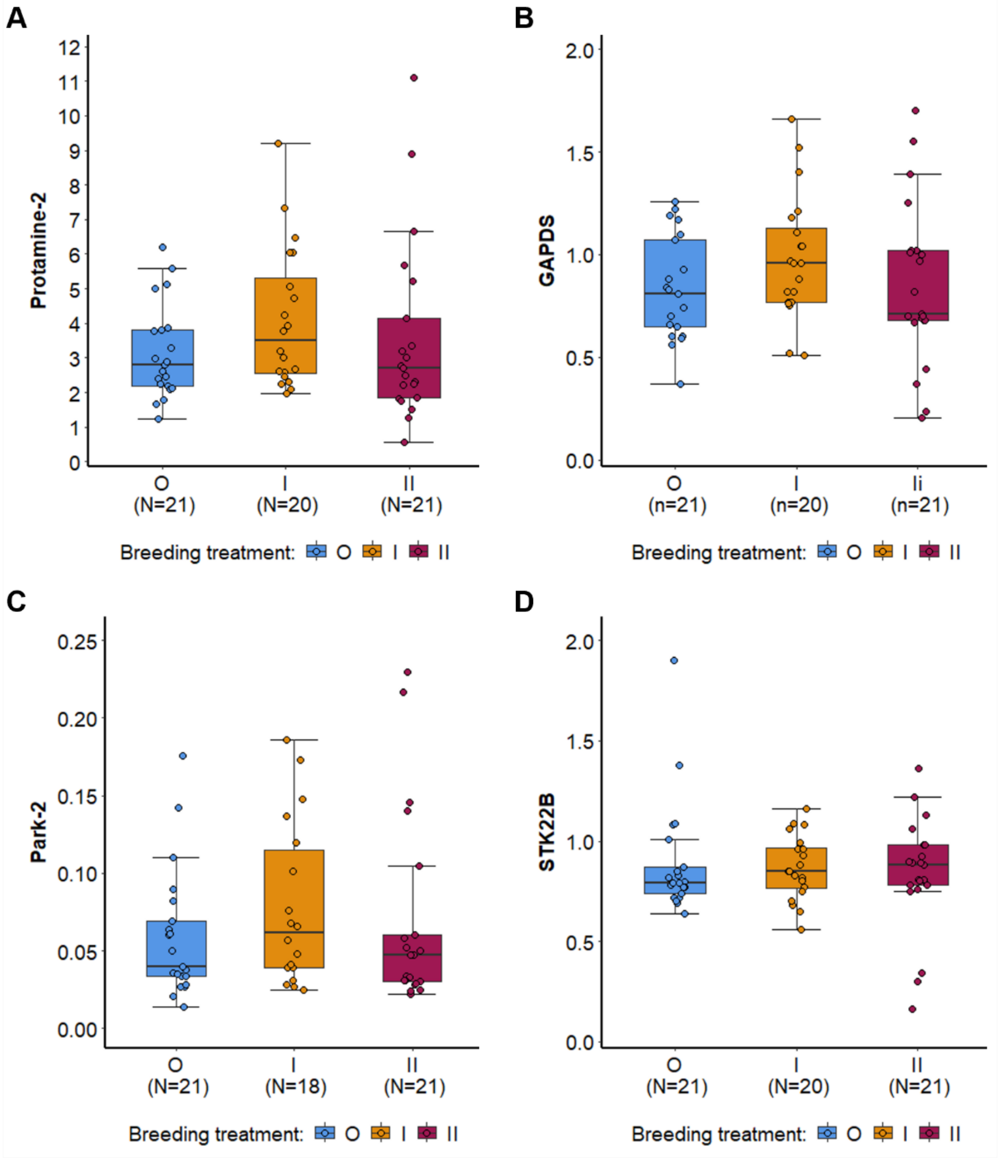

Supplementary Figure 8. Gene expression of proteins relevant for spermiogenesis depending on males' breeding treatments. Points represent raw data, boxplots show median (center line), interquartile range (IQR, box), and 1.5x IQR (whiskers) of (**A**) Protamine-2, (B) GAPDS, (C) Park-2, and (**D**) STK22B for outbred (O, blue), 1st generation inbred (I, orange) and 2nd generation inbred (II, red) males (shown from left to right, respectively).

There was no significant correlation between gene expression and sperm concentration, motility or sperm velocity parameters at T0, respectively, when pooling the three treatment groups. However, when analyzing the groups separately, we found a negative correlation between sperm concentration and GAPDS in I mice (I: rs= -0.492, p=0.027, Table S9, Fig. S9A) and a positive correlation between sperm concentration and Protamine-2 in II mice (II: rs= 0.438, p=0.047, Table S9, Fig. S9B), however this correlation was not significant after FDR correction and there was no significant difference in the slopes between the three treatment groups (Fig. S9A and B). Furthermore, the percentage of motile sperm was negatively correlated with GAPDS (I: rs= -0.452, p=0.046, Table S9, Fig. S9C) and Park-2 (I: rs= -0.519, p=0.027, Table S9, Fig. S9D) in I mice and with Protamine-2 (O: rs= -0.455, p=0.038, Table S9, Fig. S9E) in O mice, and tended to be positively correlated with Protamine-2 (II: rs= 0.432, p=0.051, Table S9, Fig. S9E) and GAPDS (II: rs= 0.405, p=0.068, Table S9, Fig. S9C) in II mice, though all were not significant after FDR correction (Table S9). Additionally, for the correlations of gene expression (GAPDS, Park-2 and Protamine-2) versus the percentage of motile sperm, the slope of II males differed significantly from the slope of O and I males. (GLM: GAPDS vs %Motile: F=6.925, p=0.002, II vs O: T=-2.815, p=0.007, II vs. I: T=-3.506, p=0.001 (Fig. S9C); Park-2 vs % Motile: F=3.267, p=0.046, II vs O: T=-0.578, p=0.566, II vs. I: T=-2.462, p=0.017 (Fig. S9D); Protamine-2 vs %Motile: F=6.418, p=0.003, II vs O: T=-2.941, p=0.005, II vs. I: T=-3.231, p=0.002 (Fig. S9E)).

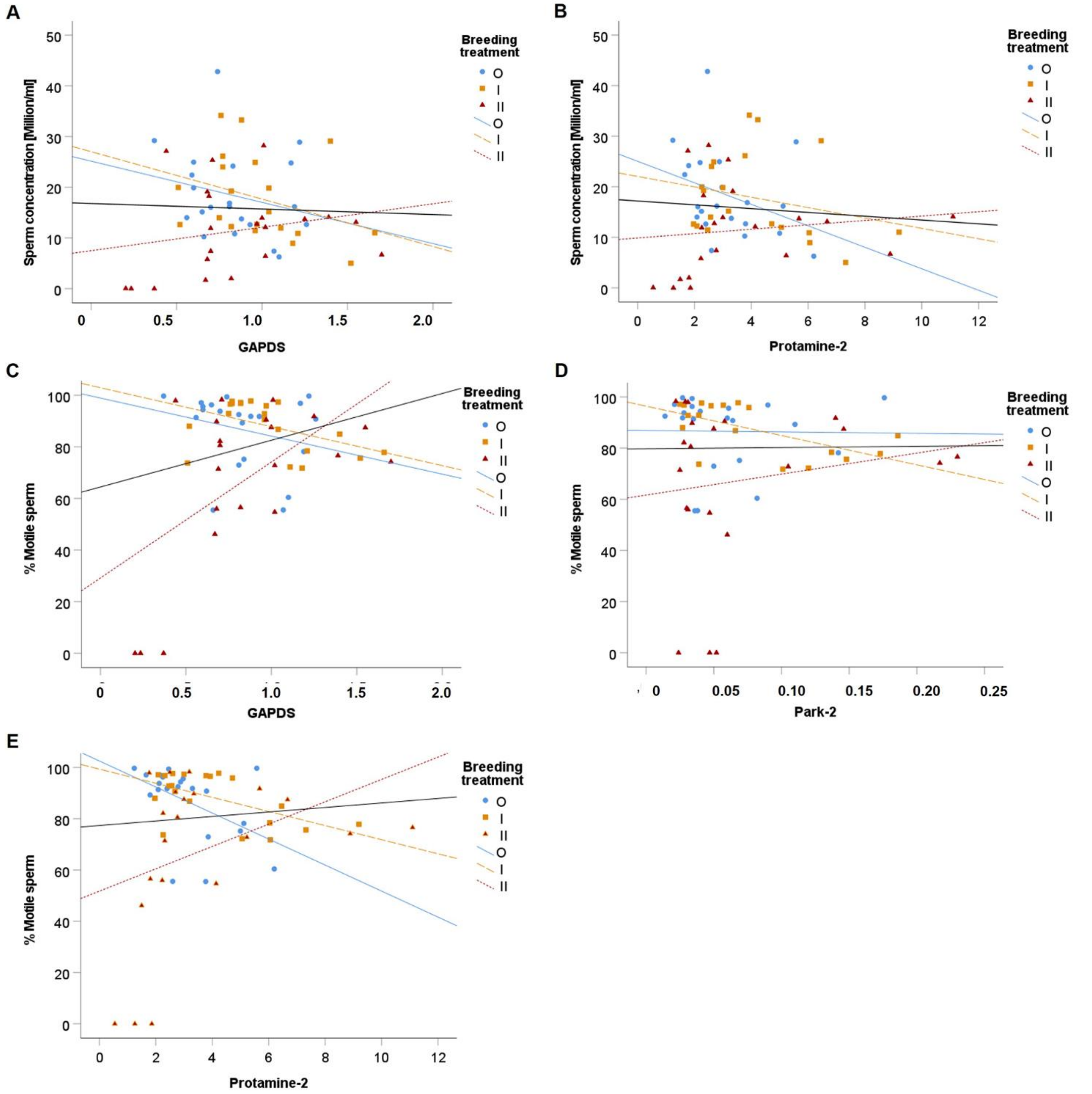

Supplementary Figure 9. Correlation between gene expression of proteins associated with spermiogenesis and sperm parameters measured at T0 (initial measurement). Symbols and trendlines represent outbred (O, blue circles, solid line), 1st generation inbred (I, orange squares, dashed line) and 2nd generation inbred (II, red triangles, dotted line) males. The black line represents the overall correlation. Graphs present correlations between sperm concentration and (**A**) GAPDS or (**B**) Protamine-2 and correlations between percentage of motile sperm and (**C**) GAPDS, (**D**) Park-2, and (**E**) Protamine-2.

2.2.2 Correlations between sperm traits and reproductive organs

We tested whether the sperm concentration was correlated with the reproductive organs. When analyzing all males, both the relative testes mass and relative epididymal mass were positively correlated with the sperm concentration measured at T0 (relative testes mass: rs= 0.405, p=0.001, FDR adjusted p=0.002; relative epididymal mass: rs= 0.410, p=0.001, FDR adjusted p=0.002, Table S9, Fig. S10A and B). However, when analyzing the 3 groups separately, this correlation was only present in II mice for both parameters. Thus, the slope of the correlation between sperm concentration and relative testes mass of II mice was significantly different from O and I mice (GLM: II vs O: T=-2.0, p=0.05, II vs I: T=-2.575, p=0.013). Additionally, the sperm concentration was correlated with the relative anogenital distance when pooling all mice (rs= 0.265, p=0.037, Table S9, Fig. S10C), though not after FDR correction and with no differences in the slopes. In outbred mice, we also found a negative correlation between sperm concentration and body mass (rs= -0.524, p=0.015, FDR adjusted p=0.06, Table S9).

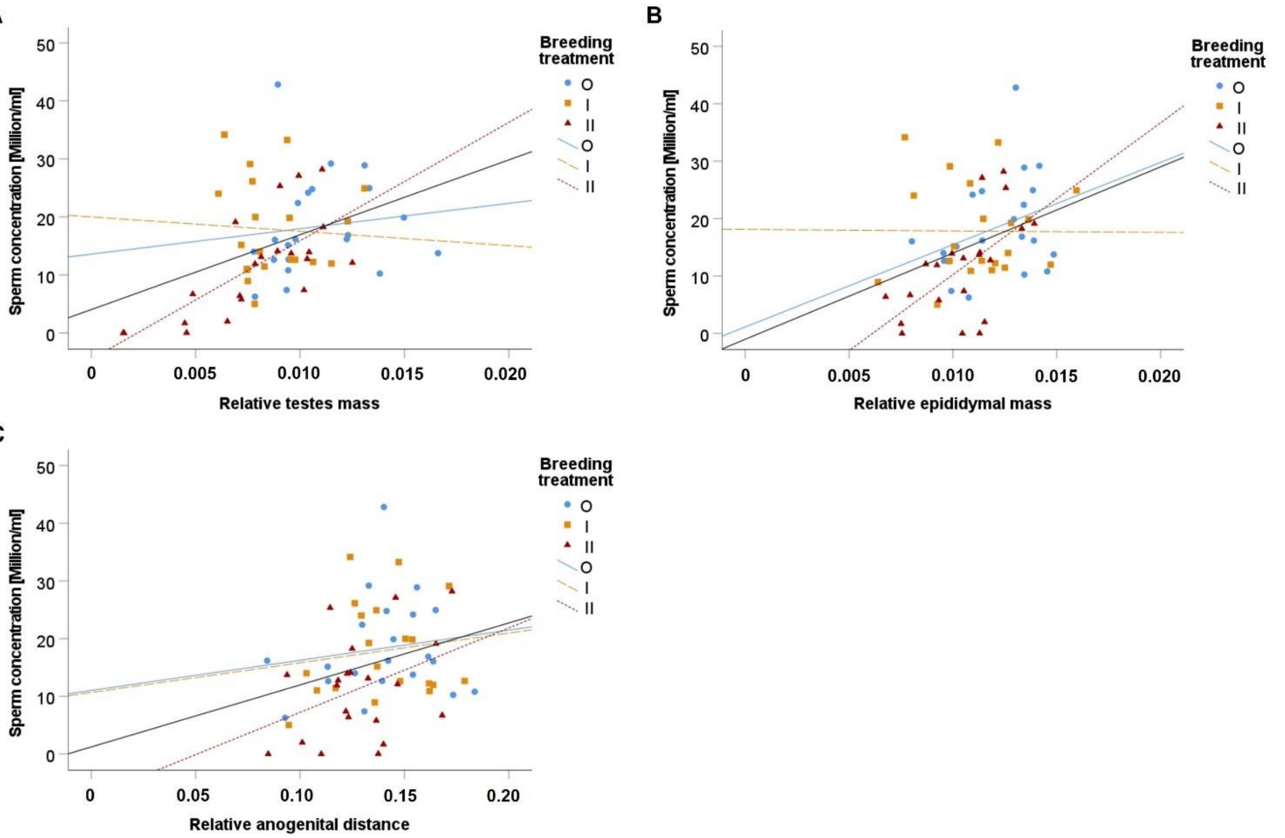

Supplementary Figure 10. Relationships between reproductive organ mass and sperm concentration. Symbols and trendlines represent outbred (O, blue circles, solid line), 1st generation inbred (I, orange squares, dashed line) and 2nd generation inbred (II, red triangles, dotted line) males. The black line represents the overall correlation. Graphs show correlations between sperm concentration (M/ml) and (**A**) relative testes mass, (**B**) relative epididymal mass, and (**C**) relative anogenital distance. Relative organ mass was calculated as total organ mass / body mass, and relative anogenital distance was calculated as anogenital distance / body length (snout to tail base).

2.3 Correlations between pre- versus post-copulatory traits

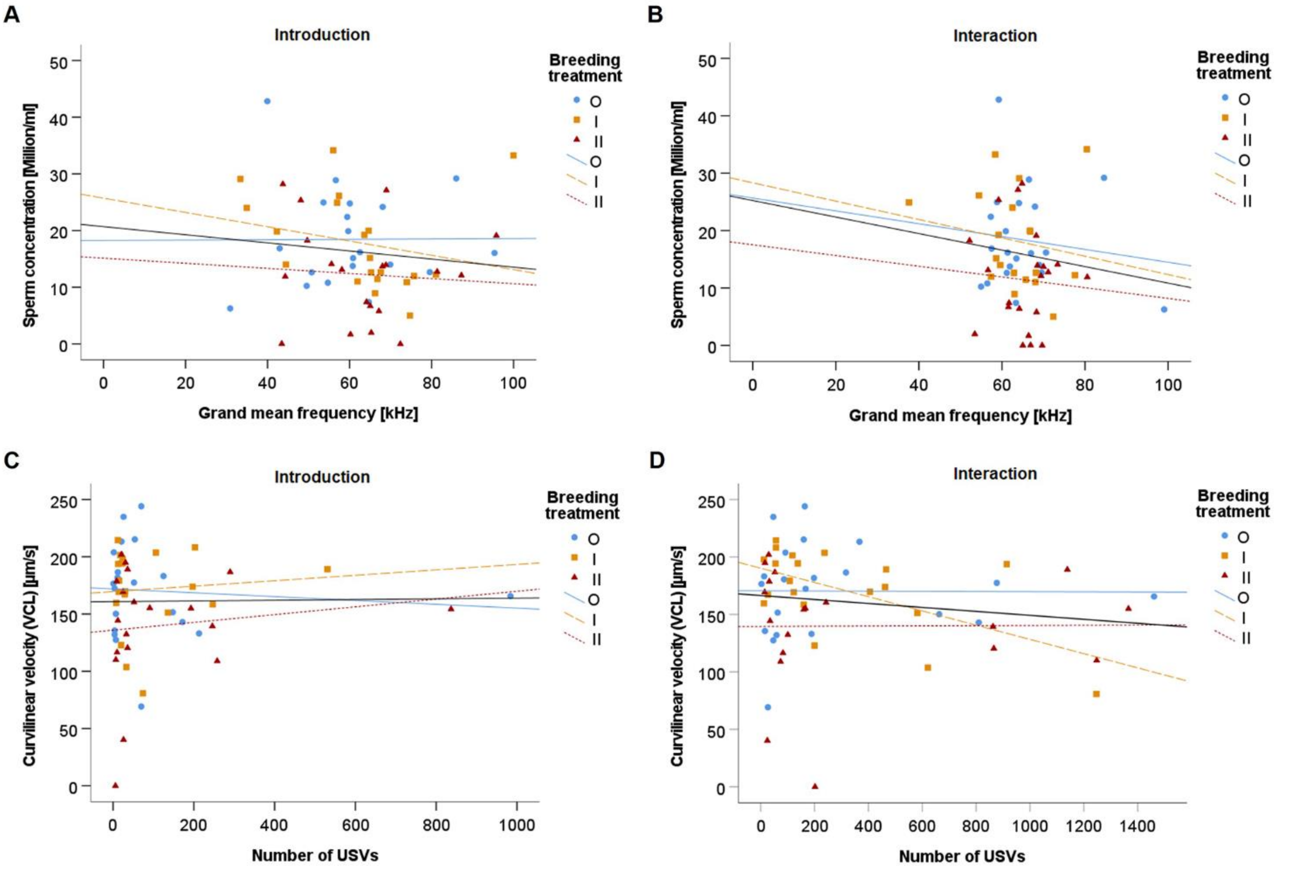

Supplementary Figure 11. Correlation between USV emission and sperm parameters. Symbols and trendlines represent outbred (O, blue circles, solid line), 1st generation inbred (I, orange squares, dashed line) and 2nd generation inbred (II, red triangles, dotted line) males. The black line represents the overall correlation. Graphs show correlations between (**A** and **B**) sperm concentration and grand mean frequency of USVs during the (**A**) introduction phase and (**B**) interaction phase and (**C** and **D**) correlation between average sperm curve speed and number of USVs emitted during the (**C**) introduction phase and (**D**) interaction phase.

3 Supplementary Tables

Supplementary Table 4. Generalized linear mixed models (GZLMM) to test the effects of recording phase (ph1: introduction phase, ph2: interaction phase) or breeding treatment (O: outbred, I: 1st generation inbred and II: 2nd generation inbred) on sonic vocalizations and USV parameters. Post-hoc pairwise comparisons were conducted using sequential Sidak method to correct for multiple testing. Significant p-values (p<0.05) are in bold, and trends (p<0.1) are in italic.

Parameter	Fixed effect	GZLMM		Post-hoc pairwise comparison		95% Confidence interval lower - upper
		F	P	Contrasts	P adjusted (sequential Sidak)	
Squeak n=63	breeding treatment	2.038	0.135	O - I	0.235	-15.974 – 2.640
				O - II	0.844	-6.163 – 5.046
				I - II	0.235	-2.789 – 15.007
	phase	76.161	**<0.001**			13.062 – 26.335
	treatment*phase	2.368	*0.098*	Ph1: O - I	0.164	-9.827 – 1.147
				Ph1: O - II	0.364	-6.305 – 1.748
				Ph1: I - II	0.425	-3.035 – 7.158
				Ph2: O - I	0.506	-27.432 – 13.599
				Ph2: O - II	0.424	-8.377 – 26.564
				Ph2: I - II	0.229	-6.167 – 38.187
MFVs n=63	breeding treatment	0.351	0.705	O - I	0.815	-4.181 – 2.421
				O - II	0.855	-2.307 – 2.779
				I - II	0.815	-2.297 – 4.529
	phase	16.606	**<0.001**			-5.785 – -1.506
	treatment*phase	0.689	0.504	Ph1: O - I	0.91	-6.253 – 5.575
				Ph1: O - II	0.748	-3.997 – 8.100
				Ph1: I - II	0.748	-4.022 – 8.803
				Ph2: O - I	0.748	-3.697 – 1.688
				Ph2: O - II	0.782	-3.046 – 1.727
				Ph2: I - II	0.782	-2.048 – 2.739
USV count n=63	breeding treatment	0.427	0.654	O - I	0.818	-95.160 – 57.206
				O - II	0.754	-121.025 – 55.811
				I - II	0.818	-96.432 – 69.172
	phase	49.895	**<0.001**			73.960 – 177.071
	treatment*phase	0.072	0.931	Ph1: O - I	0.821	-61.084 – 36.893
				Ph1: O - II	0.748	-80.315 – 36.669
				Ph1: I - II	0.821	-65.660 – 46.204
				Ph2: O - I	0.897	-184.702 – 127.540
				Ph2: O - II	0.895	-222.507 – 130.399

				Ph2: I - II	0.897	-181.164 – 146.219
Vocal repertoire n=63	breeding treatment	1.373	0.257	O - I	0.549	-1.112 – 0.594
				O - II	0.284	-1.797 – 0.350
				I - II	0.516	-1.485 – 0.556
	phase	23.114	**<0.001**			-2.012 – 0.855
	treatment*phase	0.332	0.725	Ph1: O - I	0.660	-1.296 – 0.824
				Ph1: O - II	0.332	-2.213 – 0.491
				Ph1: I - II	0.470	-1.908 – 0.658
				Ph2: O - I	0.855	-1.568 – 1.002
				Ph2: O - II	0.735	-1.924 – 0.862
				Ph2: I - II	0.855	-1.536 – 1.041
Log (mean USV length) n=62	breeding treatment	0.885	0.415	O - I	0.747	-0.092 – 0.168
				O - II	0.747	-0.172 – 0.093
				I - II	0.46	-0.220 – 0.064
	phase	55.423	**<0.001**			0.202 – 0.349
	treatment*phase	0.075	0.928	Ph1: O - I	0.79	-0.134 – 0.224
				Ph1: O - II	0.79	-0.237 – 0.136
				Ph1: I - II	0.575	-0.295 – 0.104
				Ph2: O - I	0.869	-0.117 – 0.178
				Ph2: O - II	0.869	-0.176 – 0.117
				Ph2: I - II	0.735	-0.218 – 0.098
Grand mean frequency n=61	breeding treatment	0.19	0.827	O - I	0.932	-5.792 – 5.311
				O - II	0.919	-8.522 – 5.263
				I - II	0.919	-8.076 – 5.299
	phase	1.399	0.239			-1.716 – 6.806
	treatment*phase	0.399	0.672	Ph1: O - I	0.876	-13.423 – 8.395
				Ph1: O - II	0.876	-14.896 – 8.414
				Ph1: I - II	0.879	-10.144 – 8.691
				Ph2: O - I	0.834	-4.512 – 8.577
				Ph2: O - II	0.995	-5.312 – 5.277
				Ph2: I - II	0.834	-8.607 – 4.506
Log (ISI) n=61	breeding treatment	0.265	0.768	O - I	0.88	-0.050 – 0.083
				O - II	0.88	-0.049 – 0.086
				I - II	0.946	-0.052 – 0.056
	phase	0.853	0.358			-0.056 – 0.021
	treatment*phase	2.087	0.13	Ph1: O - I	0.839	-0.072 – 0.089
				Ph1: O - II	0.446	-0.044 – 0.154
				Ph1: I - II	0.446	-0.043 – 0.137
				Ph2: O - I	0.69	-0.049 – 0.099
				Ph2: O - II	0.69	-0.087 – 0.051
				Ph2: I - II	0.467	-0.122 – 0.036

Log (IBI) n=61	breeding treatment	0.927	0.399	O - I	0.491	-0.533 – 0.164
				O - II	0.495	-0.484 – 0.174
				I - II	0.83	-0.247 – 0.307
	phase	56.489	**<0.001**			-0.805 – -0.469
	treatment*phase	1.606	0.205	Ph1: O - I	0.191	-0.864 – 0.118
				Ph1: O - II	0.363	-0.733 – 0.203
				Ph1: I - II	0.573	-0.271 – 0.486
				Ph2: O - I	0.985	-0.295 – 0.302
				Ph2: O - II	0.985	0.404 – 0.315
				Ph2: I - II	0.985	-0.410 – 0.315

Supplementary Table 5. Kruskal-Wallis test on USV types comparing the 3 breeding treatments separately for both recording phases (interaction and introduction phase).

**Parameter**	**Introduction**			**Interaction**		
	**Kruskal-Wallis-H**	**n**	**P**	**Kruskal-Wallis-H**	**n**	**P**
Number of short USVs	1.513	63	0.469	1.581	63	0.454
Number of simple USVs	0.029	63	0.986	0.094	63	0.954
Number of complex USVs	4.734	63	*0.094*	0.328	63	0.849

Supplementary Table 6. Generalized Linear Mixed Models (GLMM) testing effects of breeding treatment (O: outbred, I: 1st generation inbred and II: 2nd generation inbred) on spectro-temporal features using single USV datapoints of the interaction phase. Significant p-values (p<0.05) are in bold, and trends (p<0.1) are in italic.

**Mean frequency of USVs** (GLMM using single datapoints)					Relative Likelihood
**model breeding treatment** AICc = -40255.01					1
Random effects:					
Groups	Name	Variance	Std.Dev.		
Replicate	(Intercept)	0.07277	0.2698		
Residual		0.02208	0.1486		
Number of observations: 17163, groups: Replicate, 62					
Fixed effects:					
	Estimate	Std. Error	t value	P value	
(Intercept)	2.34228	0.18888	12.401	**<0.001**	
genetic-I	0.62936	0.30652	2.053	**0.040**	
genetic-II	-0.02116	0.25261	-0,084	0.933	
**Null model AICc** = -40252.2					**0.25**
**USV length** (GLMM using single datapoints)					Relative Likelihood
**model breeding treatment** AICc = 149221.9					0.61
Random effects:					
Groups	Name	Variance	Std.Dev.		
Replicate	(Intercept)	2.2e-07	0.0005		
Residual		9.3e-03	0.0966		
Number of observations: 17636, groups: Replicate, 63					
Fixed effects:					
	Estimate	Std. Error	t value	P value	
(Intercept)	0.0022837	0.0003503	6.519	**<0.001**	
genetic-I	0.0008174	0.0004958	1.648	*0.0993*	
genetic-II	0.0003124	0.0004568	0.684	0.4939	
**Null model** AICc = 149220.9					1
**Inter-syllable interval (ISI)** (GLMM using single datapoints)					Relative Likelihood
model breeding treatment AICc = -34059.72					1
Random effects:					
Groups	Name	Variance	Std.Dev.		
Replicate	(Intercept)	0.000	0.00		
Residual		3.131	1.77		
Number of observations: 11382, groups: Replicate, 56					
Fixed effects:					
	Estimate	Std. Error	t value	P value	
(Intercept)	80.154	1.692	47.381	**<0.001**	
genetic-I	-7.977	2.294	-3.478	**0.0005**	
genetic-II	-1.929	2.193	-0.879	0.379	

**Null model** AICc = -34049.51					**0.006**
**Inter-bout interval (IBI)** (GLMM using single datapoints)					Relative Likelihood
**model breeding treatment** AICc = 20158.45					0.23
Random effects:					
Groups	Name	Variance	Std.Dev.		
Replicate	(Intercept)	0.1172	0.3424		
Residual		1.7885	1.3374		
Number of observations: 6189, groups: Replicate, 62					
Fixed effects:					
	Estimate	Std. Error	t value	P value	
(Intercept)	0.10856	0.05614	1.934	*0.0531*	
genetic-I	-0.01440	0.08035	-0.179	0.8578	
genetic-II	0.06343	0.07950	0.798	0.4249	
**Null model** AICc = 20155.51					1

Supplementary Table 7. Generalized Linear Mixed Models (GZLMM) to test the effects of sperm collection timepoint (T0: directly after sperm collection, T2: 2h after sperm collection) or breeding treatment (O: outbred, I: 1st generation inbred and II: 2nd generation inbred) on sperm parameters. Post-hoc pairwise comparisons were conducted using sequential Sidak method to correct for multiple testing. Significant p-values (p<0.05) are in bold, and trends (p<0.1) are in italic.

**Parameter**	**Fixed effect**	**GZLMM**		**Post-hoc pairwise comparison**		**95% Confidence interval lower - upper**
		**F**	**P**	**Contrasts**	**P adjusted (sequential Sidak)**	
Sperm concentration (Million/ml) n=62	breeding treatment	5.291	**0.006**	O - I	0.72	-3.941 – 5.689
				O - II	**0.01**	1.361 – 12.995
				I - II	**0.021**	0.796 – 11.812
	timepoint	7.864	**0.006**			-2.526 – -0.435
	treatment*time point	0.161	0.852	T0: O - I	0.848	-4.785 – 5.812
				T0: O - II	**0.029**	0.536 – 13.338
				T0: I - II	**0.036**	0.363 – 12.484
				T2: O - I	0.6	-3.414 – 5.882
				T2: O - II	**0.005**	1.803 – 13.034
				T2: I - II	**0.019**	0.087 – 11.501
% Motile n=62	breeding treatment	7.431	**<0.001**	O - I	0.836	-14.198 – 11.507
				O - II	**0.003**	6.280 – 35.323
				I - II	**0.003**	6.428 – 37.867

	timepoint	72.672	<**0.001**			-24.471 – -15.245
	treatment*time point	0.165	0.848	T0: O - I	0.819	-14.563 – 11.542
				T0: O - II	**0.007**	4.566 – 34.061
				T0: I - II	**0.006**	4.859 – 36.788
				T2: O - I	0.876	-16.159 – 13.798
				T2: O - II	**0.007**	5.257 – 39.324
				T2: I - II	**0.007**	5.150 – 41.792
VCL (Curvilinear velocity, µm/s) n=60	breeding treatment	3.662	**0.029**	O - I	0.934	-19.878 – 21.624
				O - II	*0.051*	-0.106 – 51.536
				I - II	*0.051*	-0.110 – 49.794
	timepoint	223.481	<**0.001**			-96.180 – -73.670
	treatment*time point	0.503	0.606	T0: O - I	0.921	-27.754 – 25.118
				T0: O - II	*0.064*	1.137 – 62.327
				T0: I - II	*0.064*	-1.367 – 64.953
				T2: O -I	0.792	-19.923 – 26.050
				T2: O - II	0.224	-7.886 – 49.796
				T2: I - II	0.26	-9.392 – 45.174
VAP (Average path velocity, µm/s) n=60	breeding treatment	4.379	**0.015**	O - I	0.852	-9.055 – 10.938
				O - II	**0.025**	1.324 – 26.215
				I - II	**0.03**	1.053 – 24.602
	timepoint	237.953	<**0.001**			-50.802 – -39.238
	treatment*time point	0.428	0.653	T0: O - I	0.905	-12.047 – 13.590
				T0: O - II	**0.038**	0.706 – 32.489
				T0: I - II	**0.038**	0.744 – 30.908
				T2: O - I	0.848	-10.384 – 12.608
				T2: O - II	0.193	-3.492 – 25.375
				T2: I - II	0.2	-3.824 – 23.482
VSL (Straight-line velocity, µm/s) n=60	breeding treatment	3.967	**0.022**	O - I	0.881	-5.388 – 6.274
				O - II	**0.037**	0.349 – 14.861
				I - II	**0.039**	0.297 – 14.027
	timepoint	275.382	<**0.001**			-38.605 – -30.370
	treatment*time point	0.697	0.5	T0: O - I	0.929	-8.585 – 9.398
				T0: O - II	*0.082*	-0.917 – 21.377
				T0: I - II	*0.082*	-0.956 – 20.604
				T2: O - I	0.875	-5.550 – 6.511
				T2: O - II	0.305	-2.601 – 12.562
				T2: I - II	0.305	-2.743 – 11.743

Supplementary Table 8. Effects of breeding treatment (O: outbred, I: 1st generation inbred and II: 2nd generation inbred) on male reproductive organs and gene expression. Relative organ mass was calculated as total organ mass / body mass. Comparisons were conducted using ANOVA (presenting F-values) or Kruskal-Wallis Test (presenting H-values), for parametric and nonparametric variables, respectively. Significant p-values (p<0.05) are in bold, and trends (p<0.1) are in italic.

Parameter	n	Test	H or F value	P	Post-hoc pairwise comparison	
					Contrasts	P adjusted (sequential Sidak)
Body mass [g]	62	Kruskal-Wallis	0.563	0.754		
Total testes mass [g]	62	ANOVA	8.869	**<0.001**	O - I	**0.022**
					O - II	**<0.001**
					I - II	0.488
Relative testes mass	62	ANOVA	8.225	**0.001**	O - I	**0.027**
					O - II	**0.001**
					I - II	0.539
Total epididymal mass [g]	62	ANOVA	3.759	**0.029**	O - I	0.550
					O - II	**0.024**
					I - II	0.362
Relative epididymal mass	62	ANOVA	2.947	*0.060*		
Total seminal vesicles mass [g]	61	Kruskal-Wallis	0.528	0.768		
Relative seminal vesicles mass	61	ANOVA	0.436	0.649		
Protamine-2	62	Kruskal-Wallis	3.026	0.220		
GAPDS	62	ANOVA	1.278	0.286		
Park-2	60	Kruskal-Wallis	1.706	0.426		
STK22B	62	Kruskal-Wallis	0.720	0.698		

Supplementary Table 9. Spearman rank correlation between sperm parameters, gene expression and reproductive organs for all mice and separately for each breeding treatment (O: outbred, I: 1st generation inbred and II: 2nd generation inbred). Relative organ mass was calculated as total organ mass / body mass. Relative anogenital distance was calculated as Anogenital distance / body length (snout to tail base). Significant p-values (p<0.05) are in bold, and trends (p<0.1) are in italic, before and after correction for multiple testing (FDR).

**Spearman correlation**		**Sperm concentration [M/ml]**				**% Motile sperm**			
	**Breeding treatment**	**r_s**	**n**	**P**	**P adjusted (FDR)**	**r_s**	**n**	**P**	**P adjusted (FDR)**
Protamine-2	All mice	-0.031	62	0.810	0.810	0.005	60	0.973	0.973
	O	-0.347	21	0.124	0.248	-0.455	21	**0.038**	*0.076*
	I	-0.230	20	0.329	0.439	-0.432	20	*0.057*	*0.076*
	II	0.438	21	**0.047**	0.188	0.432	*21*	*0.051*	*0.076*
GAPDS	All mice	-0.085	62	0.511	0.511	0.020	60	0.877	0.877
	O	-0.278	21	0.222	0.296	-0.322	21	0.155	0.207
	I	-0.492	20	**0.027**	0.108	-0.452	20	**0.046**	0.136
	II	0.355	21	0.114	0.228	0.405	*21*	*0.068*	0.136
Park-2	All mice	-0.111	60	0.399	0.684	-0.052	58	0.701	0.797
	O	-0.019	21	0.935	0.935	-0.280	21	0.219	0.438
	I	-0.227	18	0.365	0.684	-0.519	18	**0.027**	0.108
	II	-0.151	21	0.513	0.684	-0.060	21	0.797	0.797
STK22B	All mice	0.004	62	0.974	0.974	-0.239	*60*	*0.065*	0.260
	O	0.025	21	0.915	0.974	-0.129	21	0.578	0.771
	I	-0.096	20	0.686	0.974	-0.034	20	0.887	0.887
	II	0.196	21	0.395	0.974	0.149	21	0.519	0.771
Relative testes mass	All mice	0.405	62	**0.001**	**0.002**				
	O	0.360	21	0.109	0.145				
	I	-0.039	20	0.870	0.870				
	II	0.727	21	**<0.001**	**<0.001**				
Relative epididymal mass	All mice	0.41	62	**0.001**	**0.002**				
	O	0.301	21	0.184	0.245				
	I	0.045	20	0.850	0.850				
	II	0.652	21	**0.001**	**0.002**				
Relative anogenital distance	All mice	0.265	62	**0.037**	0.148				
	O	0.153	21	0.507	0.676				
	I	0.099	20	0.677	0.677				
	II	0.323	21	0.153	0.306				
	All mice	0.072	62	0.576	0.576				

Body mass [g]	O	-0.524	21	**0.015**	*0.060*
	I	0.299	20	0.201	0.402
	II	0.165	21	0.474	0.576

Supplementary Table 10. Spearman rank correlation between USV emission, sperm parameters, and reproductive organs for all mice and separately for each breeding treatment (O: outbred, I: 1st generation inbred and II: 2nd generation inbred). Relative organ mass was calculated as total organ mass / body mass. Significant p-values (p<0.05) are in bold, and trends (p<0.1) are in italic, before and after correction for multiple testing (FDR).

Spearman rank correlation	**USV count**								
	**Introduction phase**					**Interaction phase**			
	Breeding treatment	r_S	n	P	P adjusted (FDR)	r_S	n	P	P adjusted (FDR)
Sperm concentration	all mice	-0.001	62	0.993	0.993	-0.105	62	0.461	0.866
	O	0.105	21	0.651	0.868	0.07	21	0.763	0.872
	I	-0.211	20	0.371	0.866	-0.145	20	0.541	0.866
	II	0.26	21	0.255	0.866	-0.311	21	0.17	0.866
VCL	all mice	-0.01	60	0.942	0.984	-0.154	60	0.24	0.712
	O	0.005	21	0.984	0.984	0.13	21	0.575	0.767
	I	-0.147	20	0.537	0.767	-0.424	20	*0.063*	0.504
	II	0.18	19	0.461	0.767	-0.268	19	0.267	0.712
Relative epididymal mass	all mice	-0.154	62	0.234	0.416	-0.43	62	**<0.001**	**0.0072**
	O	-0.187	21	0.418	0.557	-0.466	21	**0.033**	*0.088*
	I	-0.14	20	0.556	0.635	-0.617	20	**0.004**	**0.016**
	II	0.086	21	0.721	0.721	-0.257	21	0.26	0.416
Relative testes mass	all mice	-0.082	62	0.524	0.683	-0.303	62	**0.017**	*0.068*
	O	-0.206	21	0.371	0.594	-0.0297	21	0.191	0.406
	I	-0.011	20	0.962	0.962	-0.536	20	**0.015**	*0.068*
	II	0.29	21	0.203	0.406	-0.122	21	0.598	0.683

	grand mean frequency								
	Introduction phase					Interaction phase			
	breeding treatment	r_s	n	P	P adjusted (FDR)	r_s	n	P	P adjusted (FDR)
Sperm concentration	all mice	-0.208	59	0.114	0.456	-0.178	61	0.171	0.456
	O	0.068	20	0.777	0.973	0.008	21	0.973	0.973
	I	-0.525	20	**0.018**	0.144	-0.282	19	0.241	0.482
	II	-0.037	19	0.881	0.973	-0.092	21	0.693	0.973
Relative epididymal mass	all mice	-0.167	59	0.206	0.33	-0.354	61	**0.005**	**0.04**
	O	-0.376	20	0.102	0.238	-0.352	21	0.118	0.238
	I	0.092	20	0.701	0.748	-0.37	19	0.119	0.238
	II	-0.079	19	0.748	0.748	-0.0243	21	0.289	0.385

	mean USV length								
	Introduction phase					Interaction phase			
	breeding treatment	r_s	n	P	P adjusted (FDR)	r_s	n	P	P adjusted (FDR)
Relative epididymal mass	all mice	-0.212	60	0.104	0.208	-0.37	62	**0.003**	**0.024**
	O	-0.042	21	0.858	0.858	-0.406	21	*0.067*	0.179
	I	-0.226	20	0.339	0.387	-0.472	20	0.036	0.144
	II	-0.309	19	0.198	0.264	-0.296	21	0.192	0.264

4 References

1. Nicolakis D, Marconi MA, Zala SM, Penn DJ. Ultrasonic vocalizations in house mice depend upon genetic relatedness of mating partners and correlate with subsequent reproductive success. Front Zool. 2020;17:10.

2. Balhorn R. The protamine family of sperm nuclear proteins. Genome Biology. 2007;8(9):227.

3. Miki K, Qu W, Goulding EH, Willis WD, Bunch DO, Strader LF, et al. Glyceraldehyde 3-phosphate dehydrogenase-S, a sperm-specific glycolytic enzyme, is required for sperm motility and male fertility. Proc Natl Acad Sci U S A. 2004;101(47):16501-6.

4. Shang P, Baarends WM, Hoogerbrugge J, Ooms MP, van Cappellen WA, de Jong AA, et al. Functional transformation of the chromatoid body in mouse spermatids requires testis-specific serine/threonine kinases. J Cell Sci. 2010;123(Pt 3):331-9.

5. Song WH, Ballard JW, Yi YJ, Sutovsky P. Regulation of mitochondrial genome inheritance by autophagy and ubiquitin-proteasome system: implications for health, fitness, and fertility. Biomed Res Int. 2014;2014:981867.

6. Huggett JF. The Digital MIQE Guidelines Update: Minimum Information for Publication of Quantitative Digital PCR Experiments for 2020. Clin Chem. 2020;66(8):1012-29.
